# Amino Acid Residues Controlling Domain Interaction and Interdomain Electron Transfer in Cellobiose Dehydrogenase

**DOI:** 10.1002/cbic.202300431

**Published:** 2023-09-28

**Authors:** Bettina Motycka, Florian Csarman, Melanie Rupp, Karoline Schnabel, Gabor Nagy, Kwankao Karnpakdee, Stefan Scheiblbrandner, Rupert Tscheliessnig, Chris Oostenbrink, Michal Hammel, Roland Ludwig

**Affiliations:** ^1^ University of Natural Resources and Life Sciences Vienna Department of Food Science and Technology Institute of Food Technology Muthgasse 18 1190 Vienna Austria; ^2^ University of Natural Resources and Life Sciences, Vienna Department of Biotechnology Institute of Bioprocess Science and Engineering Muthgasse 18 1190 Vienna Austria; ^3^ Molecular Biophysics and Integrated Bioimaging Lawrence Berkeley National Laboratory Cyclotron road 1 94720 Berkeley California USA; ^4^ Max Planck Institut für Multidisciplinary Sciences Department of Theoretical and Computational Biophysics Am Fassberg 11 37077 Göttingen Germany; ^5^ Division of Biophysics Gottfried-Schatz-Research-Center Medical University of Graz Neue Stiftingtalstraße 6 8010 Graz Austria; ^6^ University of Natural Resources and Life Sciences Vienna Department of Material Sciences and Process Engineering Institute of Molecular Modeling and Simulation Muthgasse 18 1190 Vienna Austria

**Keywords:** cellobiose dehydrogenase, electron transfer, multistate modeling, molecular dynamic simulation, small angle X-ray scattering

## Abstract

The function of cellobiose dehydrogenase (CDH) in biosensors, biofuel cells, and as a physiological redox partner of lytic polysaccharide monooxygenase (LPMO) is based on its role as an electron donor. Before donating electrons to LPMO or electrodes, an interdomain electron transfer from the catalytic FAD‐containing dehydrogenase domain to the electron shuttling cytochrome domain of CDH is required. This study investigates the role of two crucial amino acids located at the dehydrogenase domain on domain interaction and interdomain electron transfer by structure‐based engineering. The electron transfer kinetics of wild‐type *Myriococcum thermophilum* CDH and its variants M309A, R698S, and M309A/R698S were analyzed by stopped‐flow spectrophotometry and structural effects were studied by small‐angle X‐ray scattering. The data show that R698 is essential to pull the cytochrome domain close to the dehydrogenase domain and orient the heme propionate group towards the FAD, while M309 is an integral part of the electron transfer pathway – its mutation reducing the interdomain electron transfer 10‐fold. Structural models and molecular dynamics simulations pinpoint the action of these two residues on the domain interaction and interdomain electron transfer.

## Graphical abstract

Cellobiose dehydrogenase serves as an auxiliary enzyme donating electrons to lytic polysaccharide monooxygenase in biomass depolymerization, as well as a biorecognition element in biosensors due to its electron transfer capability. The involvement of two amino acids in the interdomain electron transfer process is investigated in depth with stopped‐flow spectrophotometry, small‐angle X‐ray scattering, multistate modeling, and molecular dynamics simulations.

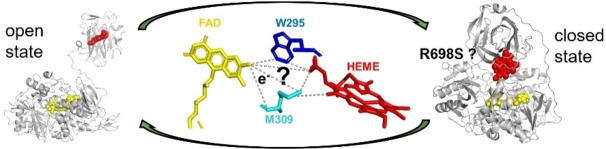

## Introduction

**Figure 1:**
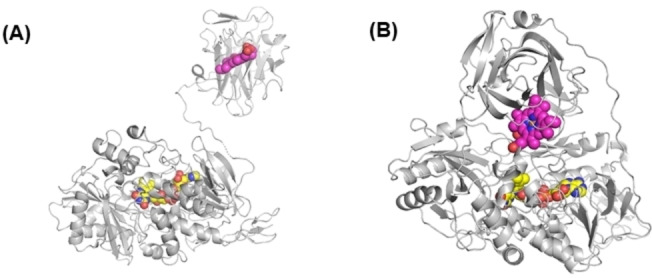
Models of the open state (4QI7, **A**) and the closed state (4QI6, **B**) of CDH. The FAD in the DH domain is shown in yellow, and the heme *b* in the CYT domain is red.

Plant biomass conversion into renewable materials and building blocks is limited by the complex, interconnected structure of plant biomass as well as the resistance of its polymers towards depolymerization.[^1^, ^2^] For this reason, the application of powerful oxidoreductases for efficient polysaccharide depolymerization processes becomes increasingly prominent. Especially lytic polysaccharide monooxygenases (LPMOs)[^3^, ^4^] are active on a wide variety of carbohydrate polymers as substrates, support the action of hydrolytic enzymes, and degrade recalcitrant plant polymers. LPMOs enhance the degradation of cellulose and other polysaccharides by the regioselective hydroxylation of carbon atoms and thereby induced cleavage of the glycosidic bond.[^5^, ^6^] LPMOs depend on auxiliary enzymes to provide hydrogen peroxide as cosubstrate^[7]^ and reductants such as diphenols, ascorbates or cellobiose dehydrogenase (CDH)[^8^, ^9^] as electron donors. The dehydrogenase domain of CDH is a member of the glucose‐methanol‐choline(GMC)‐oxidoreductase superfamily.[^10^, ^11^] Structural analysis of full‐length CDH by X‐ray crystallography[^12^, ^13^] and small‐angle X‐ray scattering (SAXS)[^14^, ^15^, ^16^] revealed that CDH is a multidomain protein featuring a larger catalytic FAD‐containing dehydrogenase domain (DH), which is connected via a flexible linker to a smaller, mobile heme *b*‐containing cytochrome domain (CYT). Tan et al.^[12]^ have been successful in crystallizing two full‐length CDHs, from *Neurospora crassa* (*Nc*CDH, 4QI7) crystallized in an open state with an extended protein linker between the two domains (Figure 1A), whereas CDH from *Myriococcum thermophilum* (syn. *Crassicarpon hotsonii*, syn. *Thermothelomyces myriococcoides, Mt*CDH, 4QI6) crystallized in a compact form (closed state) with the CYT domain in contact with the DH domain (Figure 1B). The presence and orientation of the two cofactors, FAD and heme *b*, are essential for CDH to fulfil its function as an electron transferring enzyme. Electrons are abstracted from the substrate at the DH domain by the oxidation of cellobiose to cellobiono‐1,5‐lactone which reduces FAD to FADH_2_. Then a subsequent interdomain electron transfer (IET) from the FAD (33±5 mV vs. SHE^[17]^) to the heme *b* (102±4 mV vs. SHE^[17]^) occurs. IET is followed by direct electron transfer of a single electron to an external electron acceptor such as LPMO, or *in vitro*, to an electrode. To allow the heme *b* to interact with the terminal electron acceptor, the CYT domain has to move apart from the DH domain. A flexible CYT domain that alternates between the closed and open state of CDH (Figure 1) is needed to enable its electron shuttling function. As the IET demands a small edge‐to‐edge distance between the two cofactors, CDH has to be able to adopt a closed form bringing the two cofactors in close proximity.^[18]^ A distance of 0.9 nm is found in the structure of *Mt*CDH (Figure 1B).

IET plays a crucial role in the enzymes′ natural function and technical application as it is often considered to be the rate limiting step of catalysis, but its precise mechanism is still unknown. Several researchers have studied the interface between the two domains and the IET.[^12^, ^17^, ^19^, ^20^, ^21^, ^22^] It is known from previous reports that pH and bivalent cations, stabilizing the closed form, have an influence on the CDH conformation and are hence important for the understanding of electron transfer.[^23^, ^24^, ^25^] Studying CDH from *Phanerochaete chrysosporium*, Igarashi et al., proved that both cofactors are clearly involved in IET.^[19]^ Later, Felice et al. showed on chimeric *Nc*CDH enzyme variants that steric and electrostatic interface complementarity, as well as the linker length affected IET, while the redox potential differences of two tested CYT heme *b* cofactors had no influence.^[17]^ The oriented immobilization of *Mt*CDH on gold electrode surfaces, revealed that the CYT domain mobility is necessary for IET and the final electron transfer step to the electrode.^[21]^ On the CYT domain Y99 was identified to stabilize the closed state of *Mt*CDH.^[26]^ Several amino acid residues in the DH domain with potential impact on the IET rate could be identified by Tan et al., based on the elucidated structure of *Mt*CDH and three amino acids have been shown to be strongly involved in the IET, as their mutation reduced the IET at least 10‐fold (S298Q, M309A and R698S).^[12]^ Although the details of the amino acid side chain interactions are still unclear, the formation of interactions with the propionate A of the CYT domain is suggested, which could stabilize the closed state of CDH. These previous results are the base of this work to further study IET. A better understanding would allow the optimization of CDH electron transfer properties for biosensor and biofuel cell applications.

In this study, we investigated two amino acids that have been shown to be involved in the IET of *Mt*CDH (M309 and R698^[12]^) to study their function in regard to the stabilization of the closed state and the IET. The models (Figure 1) demonstrate that IET can occur in the closed state, where both cofactors have a sufficiently close edge‐to‐edge distance. For this purpose, we produced three different *Mt*CDH variants (M309A, R698S, M309A/R698S) and compared them kinetically and structurally with the wild‐type *Mt*CDH. In particular, we studied how the mutations affected the electron transfer at different pH values (3.5–7.5) and the presence of the bivalent cation Ca^2+^ (up to 100 mM CaCl_2_). We characterized *Mt*CDH and its variants in solution under different conditions performing stopped‐flow kinetic measurements and structural analysis by SAXS. Modeling was used to understand the role of the mutated amino acids on IET.

## Results and Discussion

### Selection of residues for mutagenesis

**Figure 2:**
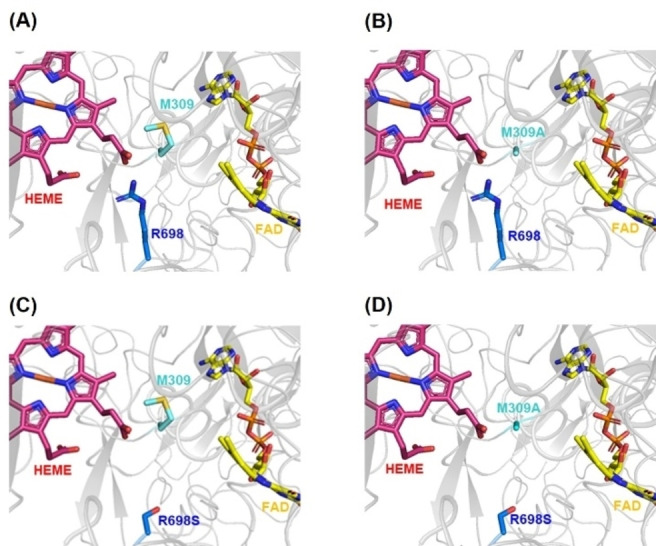
Ribbon structure of the interface between the DH and CYT domain in the closed state (4QI6) for the wild‐type *Mt*CDH**(A)**, M309A **(B)**, R698S **(C)** and M309 A/R698S **(D)**, showing the prosthetic groups (heme *b*, red; FAD, yellow, blue, orange) and the mutated side chains (M309, cyan; R698, blue) as sticks.

Amino acids that appear to be essential for the IET between the FAD and heme *b* cofactors were the focus of this study. Based on a previous study performed by Tan et al.,^[12]^ we selected M309 and R698 for a detailed investigation, since it was reported that both mutations affect IET, but their individual contribution has not been resolved. Both amino acids are located in the substrate‐binding region of the active site, between the DH and CYT domains (Figure 2A). In the previously published experiments, it was shown that the IET decreases when these positions are mutated. It remained unclear if these two amino acids are either supporting the closed state or are directly involved in the IET. To determine which role those amino acids play in facilitating the IET, we generated two single variants of *Mt*CDH: M309A (Figure 2B) and R698S (Figure 2C) and the double variant M309A/R698S (Figure 2D).

**Figure 3:**
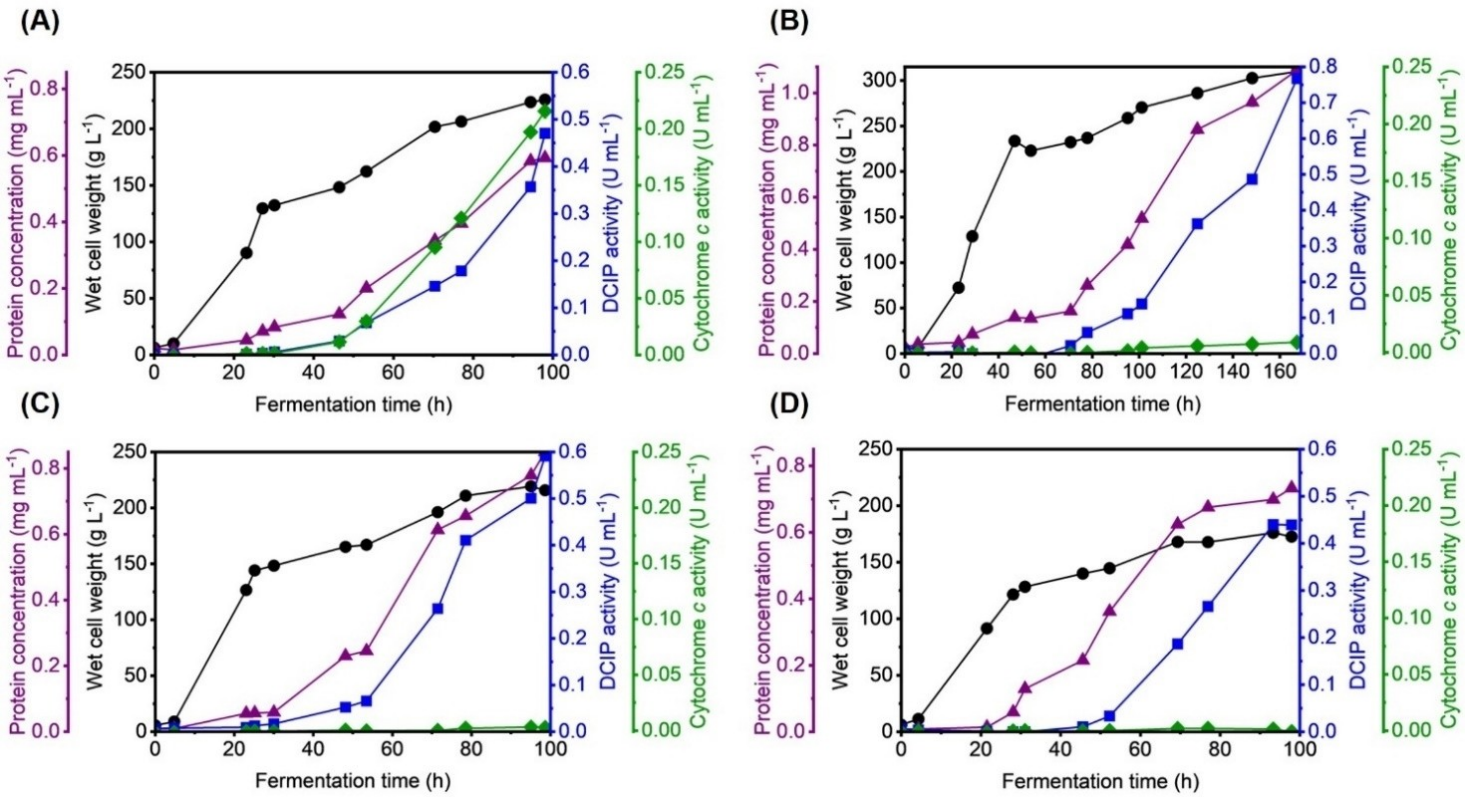
Production of wild‐type *Mt*CDH **(A)**, M309A **(B)**, R698S **(C)**, and M309A/R698S **(D)** in a 5 L bioreactor. The wet biomass (black circles), the protein concentration (purple triangles), and the activities determined using DCIP (cyan squares) or cytochrome *c* (green diamonds) as electron acceptor are shown.

All *Mt*CDH variants were recombinantly expressed in *Komagataella phaffii*. During the course of the fermentation the biomass (wet‐cell weight), the protein concentration, and the enzymatic activity were monitored (Figure 3). The fermentation time courses show a fast growth rate during the batch and glycerol fed‐batch phase. The enzyme variants were typically produced for 30 h after induction, before the fermentation process was stopped. This was the case for all variants except for M309A, where a slower initial growth phase was observed and therefore the induction was postponed, resulting in a longer overall fermentation time. In all cases the protein concentration and enzymatic activity of CDH's dehydrogenase domain measured with the DCIP assay increased constantly throughout the process. The different IET properties of the variants become obvious with the cytochrome *c* assay, which depends on the IET to transfer electrons to the CYT domain and from there to the terminal electron acceptor cytochrome *c*. For the wild‐type CDH both activities increase over time. For the variants only the DCIP activity rises, while only marginal cytochrome *c* activity could be detected. This observation confirms the influence of the introduced mutations on the IET.

To verify the purity of the produced enzymes after the purification procedure, UV/Vis spectra of the oxidized and reduced state were recorded (Figure S1) and an SDS‐PAGE (Figure S2) was performed. Both methods confirm a high purity of the enzyme preparations. During spectral measurements of the CDHs we already observed differences in the reduction of the CYT domain. For wild‐type CDH the oxidized and the reduced form of the CYT domain can be clearly distinguished, for the M3909A variant the change is much less pronounced and slower, and for the R698S and M309A/R698S almost no difference is detectable indicating a low IET rate.

### Analysis of the interdomain electron transfer

To further investigate the previous^[12]^ and initial observations, stopped‐flow measurements were performed with all purified enzymes to study the influence of pH (3.5–7.5) and Ca2+ (0–100 mM) on the reduction rates of FAD and the reduction rates of heme *b*. Previous studies showed that low pH and bivalent cations influence the domain mobility and the probability of the DH and CYT domain to form an IET‐competent closed state.[^16^, ^21^, ^25^] The closed state is supported by acidic pH, which keeps Asp and Glu residues protonated and prevents electrostatic repulsion while the addition of Ca^2+^ at less acidic pH reduces the number of negative charges at the interface or even bridges two opposing negatively charged amino acid residues.[^16^, ^23^] Using a diode array detector with the stopped‐flow apparatus allowed the combined detection of the FAD reduction rate together with the heme *b* reduction rate. The initial, catalytic step reduces FAD to FADH_2_, which is the basis of the subsequent IET step to the heme *b*. The FAD reduction rate shows the influence of the CYT domain^[23]^ on the catalytic cycle in the DH domain, while the heme *b* reduction rate is a measure of the IET between both prosthetic groups.

**Figure 4:**
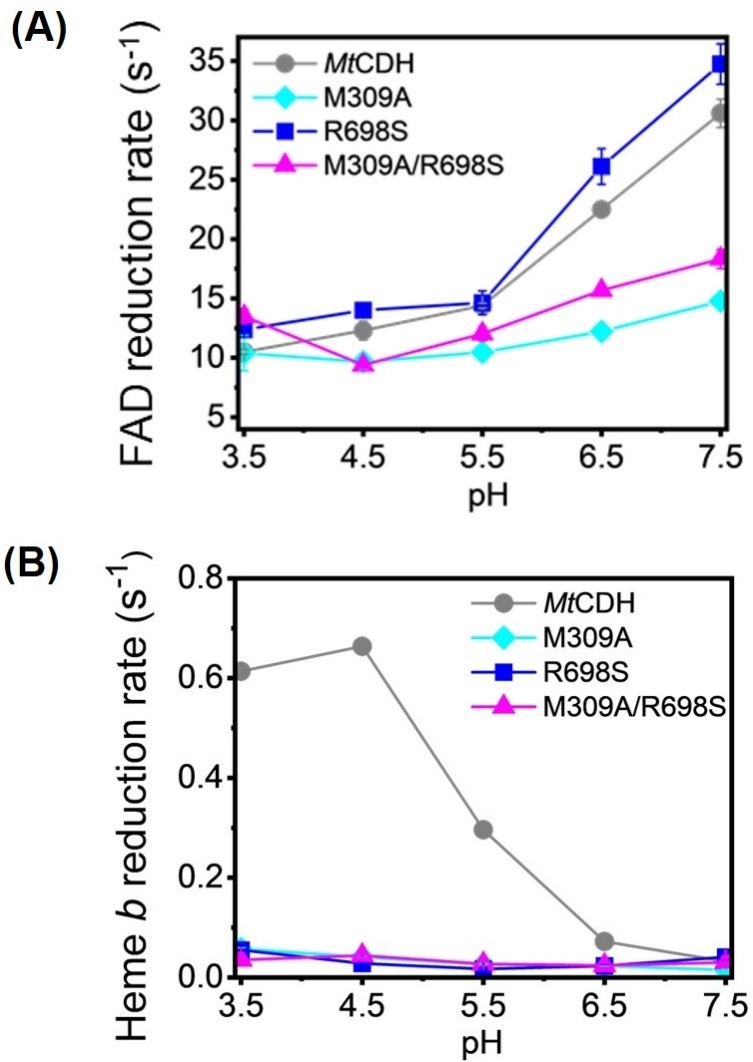
FAD (**A**) and heme *b* (**B**) reduction kinetics of wild‐type *Mt*CDH and variants at different pH values.

The FAD reduction rate in wild‐type *Mt*CDH increases with pH (Figure 4A, Table S1). A 1.4‐fold increase between pH 3.5 to 5.5 is followed by a steeper, 2.1‐fold increase from pH 5.5 to 7.5. Variant R698S shows a similar behavior and even a slightly faster FAD reduction rate than the wild‐type enzyme. Spectroelectrochemical measurements show indeed that the midpoint potential of FAD in R698S (72±5 mV vs. SHE at pH 6.0) is slightly higher than the midpoint potential of the FAD in the wild‐type *Mt*CDH (51±4 mV vs. SHE at pH 6.0), which supports an easier abstraction of electrons from the carbohydrate substrate and a faster rate. The edge‐to‐edge distance between R698 and the FAD is 0.81 nm.

Variant M309A has a similar FAD reduction rate at pH 3.5 as the wild‐type, but it barely increases with pH (1.4‐fold from pH 3.5 to 7.5). At pH 7.5 the FAD reduction rate is two‐fold lower compared to the wild‐type. The spectroelectrochemical measurement of M309A results in a midpoint redox potential of −171±5 mV vs SHE at pH 6.0. The removal of the thioether side chain, which is close to the FAD (0.75 nm) reduces the FAD midpoint redox potential by 222 mV. The greatly reduced redox potential makes the abstraction of electrons from the substrate harder and results in the reduced FAD reduction rate. The presence of R698S in the double variant M309A/R698S also seems to recover some of the pH‐dependent increase (1.5‐fold from pH 3.5 to 7.5). The low, but still statistically significant rate‐enhancing effect of the R698S mutation on the FAD reduction rate in the pH range from 6.5 to 7.5 slightly counteracts the effect of the M309A mutation. This recovery might also be found for the midpoint redox potential, which could unfortunately not be measured due to an insufficient amount of protein.

To study the IET unaffected by the catalytic step, the reduction of the FAD should be much faster than IET to not become the rate‐limiting step. In all variants the FAD reduction rate was at or above 10 s^−1^, which is at least 160 times faster than the highest observed heme *b* reduction rate in the variants (0.06 s^−1^) and therefore not a rate‐limiting step for the further investigation of the IET (Figure 4B). In comparison, wild‐type *Mt*CDH has a heme *b* reduction rate of 0.64 s^−1^ at its optimum pH of 4.5. At this pH the IET rate of the wild‐type *Mt*CDH is approximately 15 times higher than that of the variants. All introduced mutations decrease the IET strongly and no IET rates above 0.06 s^−1^ were observed for the variants. The IET rate of the wild‐type is quickly decreasing in less acidic conditions. The behavior of the wild‐type verifies that at pH 3.5–4.5 the domains are more often in the closed state, which brings the prosthetic groups in close proximity resulting in fast IET rates. Above pH 4.5 the CYT domain is more likely to be found in the open state due to electrostatic repulsion between the domains and the IET becomes less efficient.

The midpoint redox potential of the heme *b* in the wild‐type CYT measured by cyclic voltammetry is 176±2 mV vs. SHE at pH 4.5. The midpoint redox potential of the wild‐type CYT is very similar to that of the single and double variants (R698S=173±2 mV vs. SHE, M309A=170±3 mV vs. SHE, M309A/R698S=168±2 mV vs. SHE, all at pH 4.5). This shows that the mutations in the DH domain do not influence the CYT in its open state, which is the prerequisite, electrocompetent state to interact with the electrode surface during cyclic voltammetry measurements at any pH. The midpoint redox potential of the heme *b* in R698S at pH 4.5, which favors the formation of the closed state, was measured spectroelectrochemically. This method does not necessarily demand the open state of CDH to measure the redox potential. The obtained midpoint redox potential (185±4 mV vs. SHE) is only 12 mV higher than the one obtained by cyclic voltammetry and, if not caused by the different method or a high percentage of CDH molecules in the open state, indicates only a small influence of R698 on the heme *b* redox potential.

After investigating the effect of pH on the IET of the wild‐type and variant enzymes, we investigated the effect of Ca^2+^ ions as they have been shown to increase IET rates at neutral pH by increasing the domain interaction.^[21]^ This process of shielding electrostatic repulsion between the domains of CDH is not a physiological effect, as demonstrated by the high Ca^2+^ concentration (>30 mM) necessary, but is observed in many biologically important protein interactions.^[23]^ In CDH the presence of Ca^2+^ at the interface supports the closed state,[^23^, ^25^, ^27^] but likely leads to a more random interaction of the domains and less guidance to form the correct closed state. Amino acid R698, which contacts the heme propionate A in the crystal structure of *Mt*CDH is very likely involved in the stabilization of the closed state by stabilizing the heme propionate A in the correct position at the edge of the substrate channel. In absence of R698, a misorientation of the CYT domain is more likely and resulting in a lower IET rate. For that purpose, stopped‐flow measurements were performed at pH 5.5 and pH 6.5 without and with the addition of Ca^2+^ (Table S2**)**_._ In these experiments, the FAD reduction rates were also not rate limiting. A difference between the IET rates of R698S and M309A was observed. The variant R698S, which is missing the domain orientation guiding Arg‐propionate A interaction, shows a lower, only 3–4 times increased IET rate in the presence of Ca^2+^ than variant M309A. The presence of R698 results in a higher, 6 to 8‐fold increase in IET.

This result pinpoints the importance of R698 for the CYT domain orientation, especially under less acidic conditions and a more unspecific domain interaction enabled by bivalent ions.

### Analysis of the structure in solution

**Figure 5:**
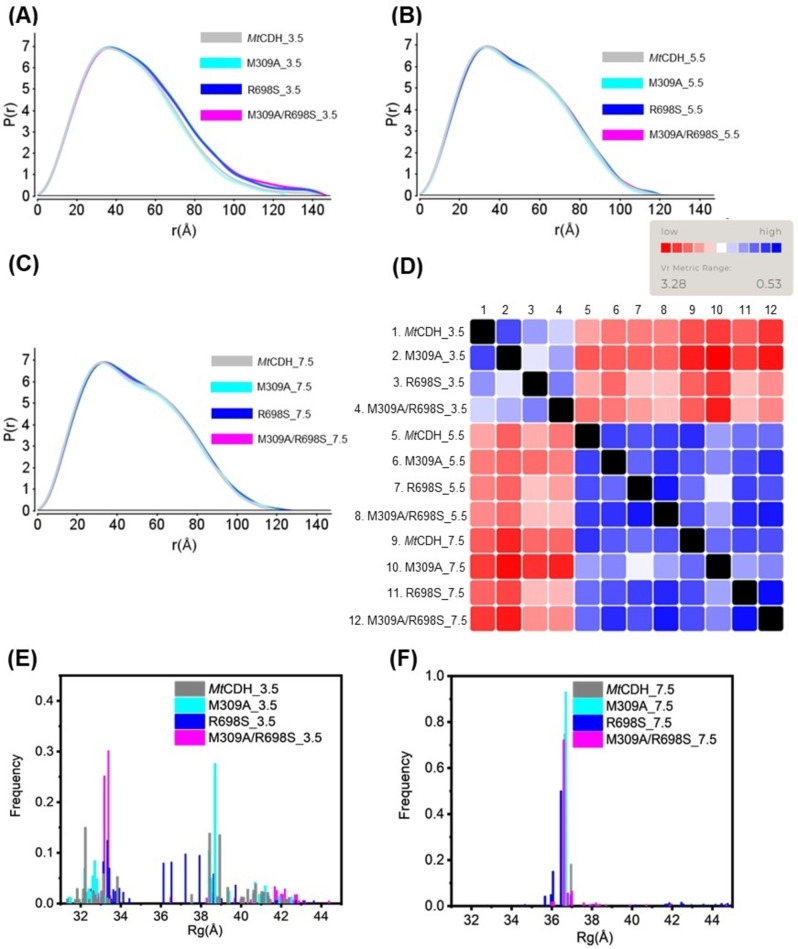
Pair‐distance‐distribution function of wild‐type *Mt*CDH and all variants obtained at pH 3.5 (**A**), 5.5 (**B**), and 7.5 (**C**), calculated from the measured data shown in Figures S3–S7. (**D**) SAXS similarity plot to compare the individual curves (red: low similarity, blue: high similarity). The results from the 50 best‐fitting combinations, consisting of two models, were used to produce the histogram for pH 3.5 (**E**) and pH 7.5 (**F**).

SAXS analysis was used to investigate the overall structure in solution under the same conditions as the kinetic studies. SAXS was performed in line with size exclusion chromatography (SEC‐SAXS) to guarantee that the scattering data were free of oligomers and aggregates. The experimental P(r) function at pH 3.5 shows a bell shape, indicating that the protein is in its closed state (Figure 5A). The shapes of the P(r) functions, which show more extended states at higher pH values are in agreement with the pH profile of the IET rates measured for MtCDH. The experimental P(r) functions of all variants showed a very high similarity to that of the wild‐type enzyme. While at pH 3.5 the P(r) function of M309A shows no significant difference to that of the wild‐type, a more open structure is observed in R698S and M309A/R698S. At pH values 5.5 and above, the P(r) functions of the variants are much more similar to the wild‐type (Figures 5B, C). This observation is also illustrated by a similarity plot (Figure 5D) of each SAXS curve. The plot demonstrates that the curves obtained at the same pH value are similar, but there are differences between low and high pH values indicating differences in the CYT domain mobility. In response to pH, the variants CYT domain adopt similar mobility to the wild‐type (Table S3). To further compare the CYT mobility in variants and wild‐type, SAXS‐based atomistic modeling of the full‐length CDH was performed. It is evident that the SAXS data are clearly better represented by a two‐state model with a mobile CYT domain than a single conformer for all measured pH values (Table S4). This implies that CDH does not remain in only one state, as is indicated by the Rg values and the conformer weights of the best combinations of two models. To simplify the interpretation, we analyzed the Rg values of the top 50 selected two‐state models. The histogram of the models′ Rg values for pH 3.5 (Figure 5E) and pH 7.5 (Figure 5F) shows that the selected conformations are similar. As shown by experimental P(r) functions (Figure 5A), the wild‐type and M309 A variants are more compact at pH 3.5 than the other variants.

The barely observed differences in the experimental P(r)'s at higher pH values are also represented in the Rg histogram (Figure 5F), showing restrained mobility of the CYT domain, as all model predictions are close to each other (Table S4). These data suggest that although IET is disrupted in the variants, the formation of the closed state at low pH is affected by R698, but not affected by M309.

To further investigate this observation, SAXS was performed in the presence and absence of Ca^2+^ at pH 5.5 (Figure S11, Tables S6, S7) and pH 6.5 (Figure S15, Tables S8, S9). The P(r) functions show a similar shape, indicating similar overall structures as further confirmed by atomistic modelling. These findings support the observation that the mutations have little influence on the overall structure.

### Molecular dynamics simulations

**Figure 6:**
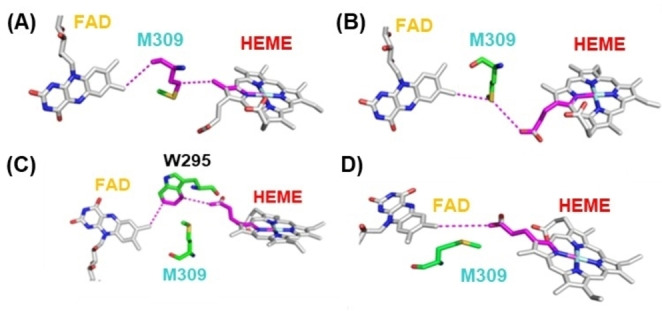
Most prominent electron transfer paths observed during the simulations: FAD‐M309‐ Heme (methylene) **(A)**, FAD‐M309‐Heme (propionate A) **(B)**, FAD‐W295‐Heme (propionate A) **(C)** and direct FAD‐Heme **(D)** pathway.

Stopped‐flow kinetic data clearly demonstrated that mutating M309 and R698 greatly decrease the IET although all enzyme variants are able to adopt a closed state shown by SAXS measurements. In order to assess the influence of the two amino acid residues on the IET, but not domain movement, a molecular dynamics (MD) simulation was performed on *Mt*CDH in the closed state (PDB ID 4QI6), to study the potential IET pathways between the FAD and heme *b* cofactor. The epath program computes the most efficient electron pathway by separately considering electron jumps through space, through hydrogen bonds, or over covalent bonds.^[28]^ Three dominant electron transfer pathways were observed during the MD simulations (Figure 6A–C). Two pathways involve residue M309, either from the FAD via the M309 side chain to the methylene group on the heme (0.69 nm, Figure 6A), or via the M309 S‐atom to the propionate of the heme (0.40 nm, Figure 6B). Jointly, these pathways are observed in 53.8 % of the analyzed conformations. The third pathway involves residue W295, in which the electron passes from FAD, through the aromatic ring of W295, to the heme propionate A (Figure 6C). A direct ET pathway (Figure 6D) only becomes favorable when conformational changes of both the surrounding protein side chains and the propionate group of the heme take place. In the wild‐type CDH, this is hindered by the sidechain of W295, and this pathway was only observed in additional simulations of a W295A variant.

The three alternative electron transfer pathways involving two different amino acids and one may conclude that the actual electron transfer in the closed state is quite robust. This is supported by the previously published W295A variant for which no reduction in the IET rate was observed.^[12]^ The rate‐limiting step in the IET process is likely the opening and closing of the CDH structure, but such large‐scale domain motions are not expected to occur in the short timescale of this simulation (20 ns). The analysis of the electron pathways confirmed the importance of M309. Direct ET between the two cofactors is unlikely due to steric hindrances at the domain interface. This highlights the importance of M309 and explains why the heme *b* reduction was reduced 15‐fold for the M309A mutant, as the primary IET pathway is absent in this variant.

The other side chain, R698, is not directly involved in the IET pathway. In the X‐ray structure of *Mt*CDH,^[12]^ it is seen to interact with the heme *b* propionate. In the MD simulations, the shortest distance between R698 and any atom of the heme *b* is on average 0.36 nm, with a maximum distance of 0.48 nm. For 86 % of the time, this shortest distance remains below 0.4 nm, suggesting that this salt bridge is stably maintained. Furthermore, R698 is involved in hydrogen bonds to the sidechains of Y99 and Q308 (directly neighboring M309) for 97 and 100 % of the simulation time, respectively, contributing to the stability of the local arrangement of amino acids. This suggests that R698 is required to correctly orient the two domains for IET and more specifically to set the orientation of M309 relative to the heme *b* propionate. This may be supported by the results of the stopped‐flow measurements with Ca^2+^. Ca^2+^ stabilizes the closed conformation at higher pH values, as the negative surface charge is compensated, as shown for the wild‐type *Mt*CDH where the IET rates become faster but no reduction could be measured for the variants. This shows that the Ca^2+^ induced closed state is not sufficient, as it cannot replace R698 in its function to properly orient M309 in the electron transfer pathway. Further confirmation is provided by the SAXS results, which clearly show that the R698S and M309A/R698S variants are not as compact as the other variants at low pH (3.5), as the salt bridge of R698 is missing in the simulation.

## Conclusions

In this study, we investigated the influence of the two side chains at the domain interface, M309 and R698, on the interdomain electron transfer of cellobiose dehydrogenase with stopped‐flow kinetic measurements and small‐angle X‐ray scattering measurements, as well as computational modeling of the electron pathways based on molecular dynamic simulations. We were able to confirm that the two amino acids play an important role in the interdomain electron transfer and the mutation of these residues greatly diminishes the heme *b* reduction. SAXS structural measurements showed a similar pair‐distance‐distribution function at different pH values and in presence of Ca^2+^ ions of the three variants in comparison with the wild‐type enzyme. This indicates that the CYT domain movement is guided by the great number of electrostatic interactions originating from charged amino acids on the CYT and DH surfaces. Based on the combination of activity studies, small‐angle X‐ray scattering measurements and molecular dynamics simulations, we conclude that M309 acts as the primary electron transfer relay between the prosthetic groups of cellobiose dehydrogenase, and thus is a key component in the interdomain electron transfer. In contrast, R698 is likely not directly involved in the electron transfer, but stabilizes the interdomain binding interface through its interactions in the closed state of the enzyme. Further, R698 possibly orients both the propionate A group of heme *b* as well as M309 to promote the electron transfer between the two. Our Ca^2+^ dependent measurements also demonstrated, that bivalent cations can increase the closed state population of the enzyme, but cannot recover the electron transfer rates in R698 deficient mutants, which support the proposed orienting function of this residue. Overall, we demonstrated that these two amino acids are both important for the interdomain electron transfer, but they fulfill different roles. Our findings may have further application of cellobiose dehydrogenase in biosensors, or in biofuel cells in combination with other enzymes such as LPMO.

## Experimental Section

### Protein expression

The *Mt*CDH wild‐type gene (*Mt*CDH IIA: gene *cdh*, UniProt A9XK88, the CYT domain belongs to CAZy family AA8, and the DH domain to CAZy family AA3_1^[29]^) integrated in a pPICZA plasmid (Invitrogen Carlsbad, CA, USA) was used to generate all *Mt*CDH variants (M309A, R689S, M309A_R698S) by a two‐step mutagenesis approach using PCR and the restriction enzyme *Dpn*I. *E. coli* NEB‐5‐alpha (New England Biolabs, Ipswich, MA, USA) was used for vector propagation and cloning. *Pichia pastoris* X‐33 (Invitrogen, Carlsbad, CA, USA) was used for enzyme expression. Transformation was performed following the protocol of manufacturer and correct integration of the gene of interest was confirmed by colony PCR and sequencing (Mycrosynth, Balgach, Switzerland). Fed‐batch fermentation of *K. phaffii*, syn. *Pichia pastoris* X‐33 (Invitrogen) transformants of the wild‐type enzyme and all variants were performed in a 5‐L bioreactor (BioFlo 120, Eppendorf, Hamburg, Germany) following a protocol modified from the procedure reported by Sygmund et al.^[30]^ Thus, precultures were prepared by inoculating 60 mL yeast extract‐peptone‐glycerol medium (YPG; 10 g L^−1^ yeast extract, 20 g L^−1^ peptone from casein, 4 g L^−1^ glycerol) supplemented with 100 mg L^−1^ zeocin (Invitrogen) with a single colony grown on a fresh yeast extract‐peptone‐glucose medium agar plate containing 100 mg L^−1^ zeocin and incubated at 25 °C and 150 rpm. After 48 h 100 μL of the cultures were transferred to 2×160 mL YPG incubated at 29 °C and 100 rpm for another 15 h. Following the Pichia Fermentation Process Guidelines (Invitrogen) all fermentations were started with an initial volume of 3.2 L basal salt medium containing 4.5 mL L^−1^ Pichia trace metals (PTM) and 300 μL L^−1^ antifoam 204 (Sigma‐Aldrich, St Luis, MO, USA). The process parameters of the fermentations were set as follows, the temperature to 30 °C, the pH to 5.0 controlled by the addition of ammonium hydroxide (25 %), the air flow rate to 1 vvm (gas flow volume normalized by liquid volume per minute) and the stirrer to 900 rpm. As soon as the initial glycerol was consumed during the batch phase, 50 % glycerol containing 12 mL L^−1^ was constantly added to increase the biomass concentration. After the initial growth phase using glycerol as carbon source (approximately 14 h) protein expression was induced by the addition of 0.5 % (v/v) methanol. One hour after the methanol addition the glycerol feed was stopped and the expression phase, a methanol containing 12 mL L^−1^ PTM feed, was started. At the time the cells were fully adapted to methanol a pulsed feed (12 mL methanol every hour) was started and the feeding strategy was performed until the end of the fermentation process. Samples were taken regularly and analyzed for wet biomass, protein concentration, determined by Bradford, and enzyme activity, determined by cytochrome *c* and 2,6‐dichloroindophenol (DCIP) assays,^[31]^ and plotted with Origin (OriginLab Corporation, Northampton, MA, USA). After harvesting, cells were removed by centrifugation at 6000 rpm for 45 min, the supernatants were clarified by filtration using a cellulose filter (type 14 A, Rotilabo, Carl Roth, Karlsruhe, Germany) and stored at 4 °C until further purification.

### Protein purification

All proteins were purified by a two‐step chromatographic procedure described by Tan et al..^[12]^ In brief, after ammonium sulphate was added to a final concentration of 30 % (sat.) the filtered supernatants were applied to a hydrophobic interaction chromatography PHE‐Sepharose column (Cytiva, Chicago, Illinois, USA) previously equilibrated with 50 mM sodium acetate pH 5.5 containing 30 % ammonium sulphate. After loading, the proteins were eluted within a linear gradient from 30 to 0 % ammonium sulphate in the same buffer. Fractions were pooled according to their specific activities and a buffer exchange to 10 mM HEPES pH 7.0 was performed (Vivaspin, 50 kDa cut‐off, Sartorius, Goettingen, Germany). The concentrated pools were then applied to an anion exchange chromatography Source 30Q column (Cytiva) that was equilibrated with 10 mM HEPES pH 7.0. After eluting within a salt gradient from 0 to 0.5 M NaCl the fractions were again analyzed and pooled according to activity and purity. After a buffer exchange to 50 mM sodium acetate pH 5.5 the concentrated samples were stored at −80 °C for further use.

### Spectral characterization

To verify the purity of the purified enzymes, a UV/Vis spectrum of each enzyme was recorded from 250 to 600 nm in the oxidized and reduced state using an Agilent 8543 diode array spectrometer (Agilent Technologies, Santa Clara, California, USA). Purified CDH was diluted in 50 mM sodium acetate pH 5.5 (sparged with N_2_ gas for at least 30 min) to an absorbance of approximately 1.1 at 280 nm, and separated into two cuvettes. To measure the reduced form of the enzyme, cellobiose was added to a final concentration of 1 mM (~1000‐fold molar excess), while for the oxidized form the same amount of water was added.

### Stopped‐flow spectroscopy

Stopped‐flow kinetics were measured with a SX20 stopped‐flow spectrometer (Applied Photophysics, Leatherhead, UK) equipped with a Photodiode Array detector. The reduction of 20 μM CDH with 10 mM cellobiose (final concentrations) was studied at 25 °C. For the determination of the pH‐optimum a 50 mM potassium‐phosphate‐citrate buffer was used. The effect of the addition of CaCl_2_ was investigated in a 50 mM sodium‐acetate buffer, pH 5.5 and a 50 mM HEPES‐HCl buffer pH 6.5 with and without CaCl_2_. All buffers were sparged with N_2_ gas for at least 30 min before the measurement. The redox state of the FAD was measured at 449 nm, while the heme *b* reduction at 563 nm. The FAD reduction rate is almost not affected by the interfering heme *b* spectrum. Although the isosbestic point at 449 nm used to determine the redox state of the FAD is slightly affected by the wavelength shift of the Soret band maximum from 421 to 430 nm,^[32]^ the FAD reduction rate for R698S is at pH 4.5 at least 450 times faster than the heme *b* reduction rate. The reduction rates were calculated by fitting the traces to exponential functions using the software Pro‐Data Viewer (Applied Photophysics). For the calculations of the rate constants an average of three measurements, for the addition of ions, and an average of five measurements for the pH profile was used.

### Spectroelectrochemistry

All spectroelectrochemical measurements were carried out in an optically transparent thin‐layer electrode (OTTLE) cell (Goodfellow Cambridge Ltd., Huntington, England, UK) following a protocol reported by Krondorfer et al.^[33]^ under N_2_ atmosphere in a glovebox. A standard three‐electrode setup comprising a platinum gauze electrode (BASi, West Lafayette, IN, USA) as working electrode, a platinum wire auxiliary electrode (BASi) as counter electrode and an Ag/AgCl electrode in saturated KCl (BASi) as reference electrode was used. Potentials, referenced to the standard hydrogen electrode (SHE, +242 mV), were applied across the cell with a Gamry 600+ potentiostat/galvanostat (Gamry, Warminster, PA, USA). All experiments were performed using 500 μL (1000 μM enzyme concentration) in a 100 mM phosphate buffer pH 6.0 containing 100 mM KCl in presence of various mediators. Spectral changes in the FAD reduction were monitored using an Agilent 8543 diode array spectrometer (Agilent Technologies).

### Cyclic voltammetry measurements

All cyclic voltammetry measurements of wild‐type *Mt*CDH and its variants were performed in 50 mM potassium‐phosphate‐citrate buffer, pH 4.5 containing 100 mM KCl as a supporting electrolyte by using an Autolab PGSTAT 204 (Methohm, Utrecht, The Netherlands). To eliminate dissolved oxygen, the buffer solution was sparged with N_2_ and Ar gas for at least 30 min before the measurement. CVs were measured following the three‐electrode configuration with platinum wire as counter electrode (BASi), Ag|AgCl in saturated KCl as reference electrode (BASi), and thiogycerol‐modified gold as working electrode. For all experiments 25 μL CDH (115 μM enzyme concentration) was trapped in a permselective membrane on a thiogycerol‐modified gold electrode. Three replications of CVs were scanned at 30 mV s^−1^. Data were analyzed using the NOVA 2.1 software. The observed peak potentials were converted from mV vs Ag|AgCl to mV vs SHE.

### Small angle X‐ray scattering: preparation, data collection and analysis

SEC‐SAXS data were collected at the SIBYLS beamline 12.3.1 at Advanced Light Source[^34^, ^35^] (LBNL Berkeley, CA, USA) at 1.27 Å wavelength with a Pilatus 2 M detector 2.1 m sample‐to‐detector distance, corresponding to a q range from 0.01 to 0.4 Å^−1^. The scattering vector is defined as q=4π sinθ/λ, where 2θ is the scattering angle, and λ is the X‐ray wavelength. For the experiments the same buffers as for the stopped‐flow measurements were used and a protein concentration of 80 μM. A size‐exclusion chromatography system (Agilent Technologies, KW‐803 column) was directly coupled with a SAXS flow cell and a multi‐angle light scattering (SEC‐SAXS‐MALS).^[35]^ For analyzing the data and computing the pair distance distribution function the program SCÅTTER (https://bl1231.als.lbl.gov/scatter/) was used. The SAXS frames recorded before the protein elution was used for the background subtraction. For analyzing the recorded SAXS data, pair‐distance‐distribution functions (P(r)) were calculated,^[36]^ representing the histogram of distances between pairs of points within a protein. The online tool SAXS Similarity^[37]^ was used to examine how similar the individual curves are. The molecular weight in the case of SEC‐MALS was calculated by using the software ASTRA (WYATT‐Technology, Santa Barbara, CA, USA) and BioXTAS RAW^[38]^ using Volume of Correlation (Vc)^[39]^ was used for the determination from the SAXS curves.

### Modeling

The online tool SWISS‐Model,^[40]^ was used to model linker region of *Mt*CDH that is not resolved in the crystal structure (pdb: 4QI6^[12]^). PyMOL (The PyMOL Molecular Graphics System, Version 1.2r3pre, Schrödinger, LLC) was used to visualize protein structures and mutated residues. Based on the crystal structure of *Mt*CDH, four N‐linked high‐mannose and five O‐linked glycans were modelled to the structure using the online tool CHARMM‐GUI^[41]^ (Lehigh University, Bethlehem, PA, USA) BILBOMD^[42]^ was used to accomplish the conformational sampling of the CYT mobility, followed by the selection of a two‐state model of CDH that best fits the SAXS data.^[43]^ For the initial model, we kept the domains rigid (CYT domain from amino acid 1–202 and DH domain from amino acid 226–870) and the linker from position 203 (**T**KT) to position 225 (**T**GV) as well as the mannose trees flexible. The histograms of Rg values were plotted using Origin (OriginLab).

Furthermore, the wild‐type *Mt*CDH was modelled and simulated using the GROMOS 54a8 force field.^[44]^ A molecular dynamics simulation was performed at a constant temperature of 300 K for 20 ns using the GROMOS simulation package.^[45]^ The simulation was found to be stable as judged by the atom positional root mean square deviations (RMSD), and secondary structure (DSSP). Electron transfer paths were calculated every 5 ps, by the GROMOS++ program epath,[^46^, ^47^] which estimates the most likely electron hopping pathway based on Marcus theory.^[28]^ Interdomain hydrogen bonds were determined using a geometric criterion, considering a hydrogen bond being formed if the hydrogen‐acceptor distance is less than 0.25 nm, while the donor‐hydrogen‐acceptor angle is more than 135 degrees.

## Supporting Information

The data that support the findings of this study are available in the supplementary information of this article and in addition SAXS ‐ datasets are available on Simple Scattering (simplescattering.com):

Dataset XSWB6KOE “Cellobiose Dehydrogenase ‐ wild‐type and its variants (pH range)”

Dataset XSYICU7O “Cellobiose Dehydrogenase ‐ wild‐type and its variants (addition of bivalent cations)”

## Conflict of interest

The authors declare that the research was conducted in the absence of any commercial or financial relationships that could be constructed as a potential conflict of interest.

1

## Supporting information

As a service to our authors and readers, this journal provides supporting information supplied by the authors. Such materials are peer reviewed and may be re‐organized for online delivery, but are not copy‐edited or typeset. Technical support issues arising from supporting information (other than missing files) should be addressed to the authors.

Supporting Information

## Data Availability

The data that support the findings of this study are openly available in Simple Scattering at https://www.simplescattering.com, reference number 2.
