# Supplementary material for: Amino Acid Residues Controlling Domain Interaction and Interdomain Electron Transfer in Cellobiose Dehydrogenase
Source: Chembiochem. 2023 Sep 28;24(22):e202300431. doi: 10.1002/cbic.202300431 (PMC10726044; doi:10.1002/cbic.202300431)
Supplement: Supplementary file 1 — Supporting Information [file CBIC-24-0-s001.pdf]

ChemBioChem

Supporting Information

Amino Acid Residues Controlling Domain Interaction and Interdomain Electron Transfer in Cellobiose Dehydrogenase

Bettina Motycka, Florian Csarman, Melanie Rupp, Karoline Schnabel, Gabor Nagy, Kwankao Karnpakdee, Stefan Scheiblbrandner, Rupert Tscheliessnig, Chris Oostenbrink, Michal Hammel, and Roland Ludwig*

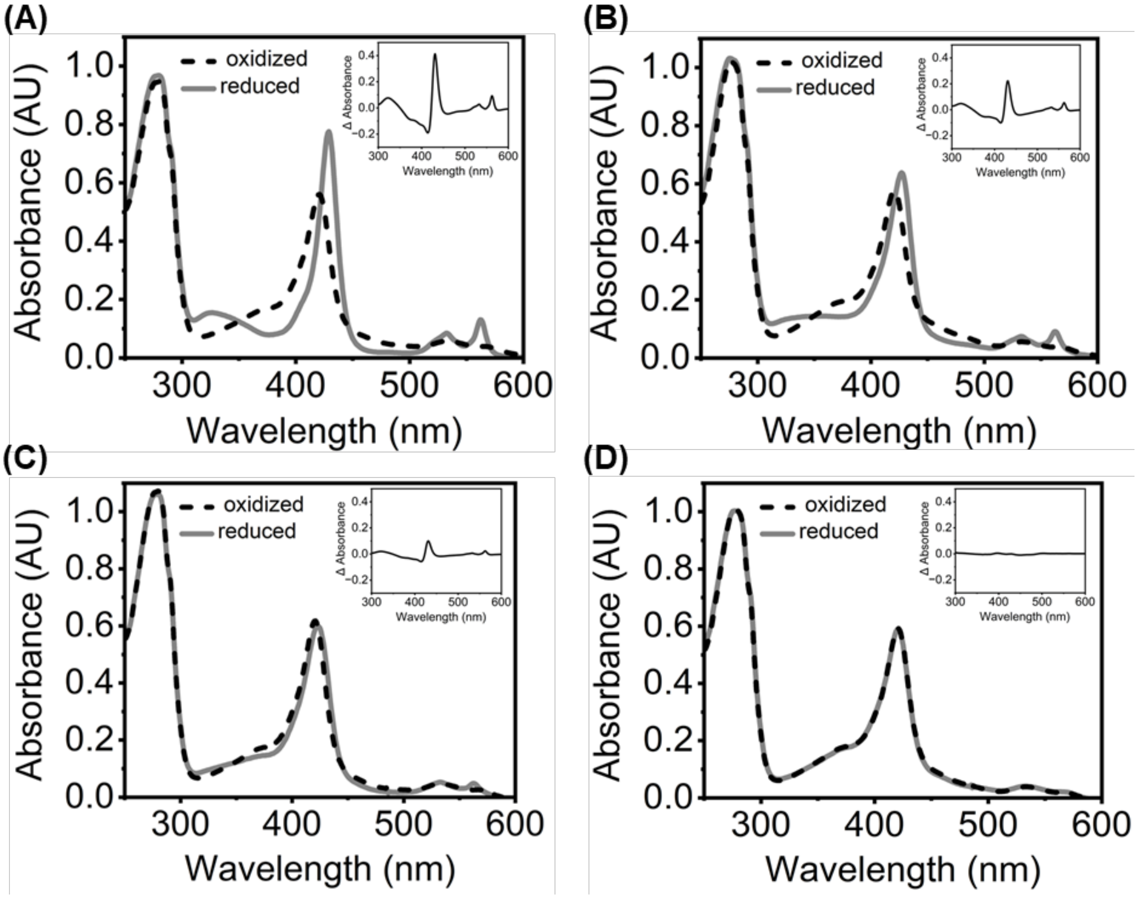

Figure S1: Spectra of oxidized and reduced wild-type *Mt*CDH **(A)**, M309A **(B)**, R698S **(C)** and M309A/R698S **(D)** in 25 mM sodium acetate buffer, pH 5.5. Cellobiose was used in excess (1 mM) to reduce the enzymes and incubated for 5 min at 30 °C before the spectra were taken. Upper right insets show the differential spectra (reduced-oxidized). The calculated purity numbers (R_Z-values, A_{420}/A_{280}) are 0.59 for the *Mt*CDH and M309A/R698S and 0.57 for M309A and R698S, respectively.

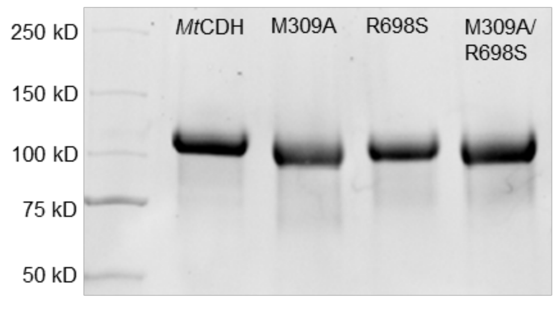

Figure S2: SDS-PAGE of wild-type *Mt*CDH and its variants, showing a high purity. The broadened bands are a result of glycosylation.

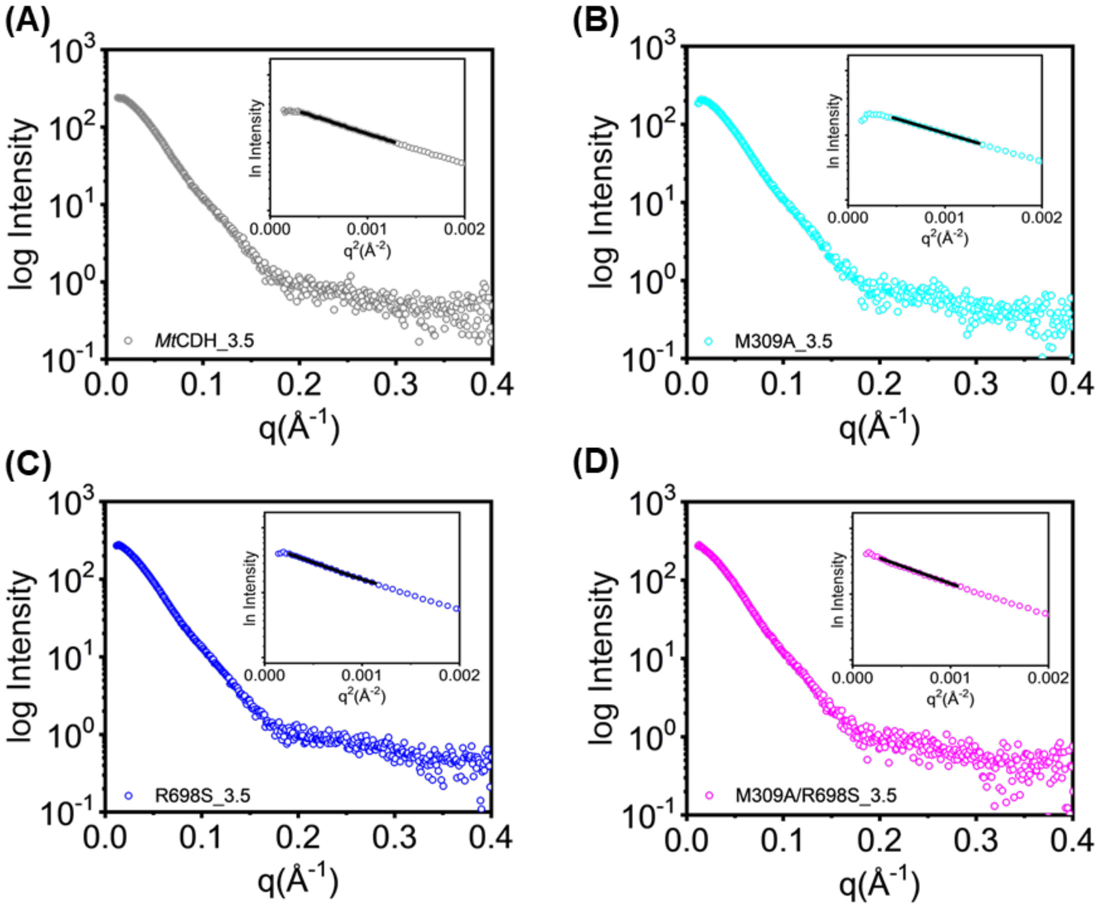

Figure S3: SAXS scattering profiles and the Guinier plots (insets) for wild-type *Mt*CDH (**A**), M309A (**B**), R698S (**C**) and M309A/R698S (**D**) at a pH of 3.5.

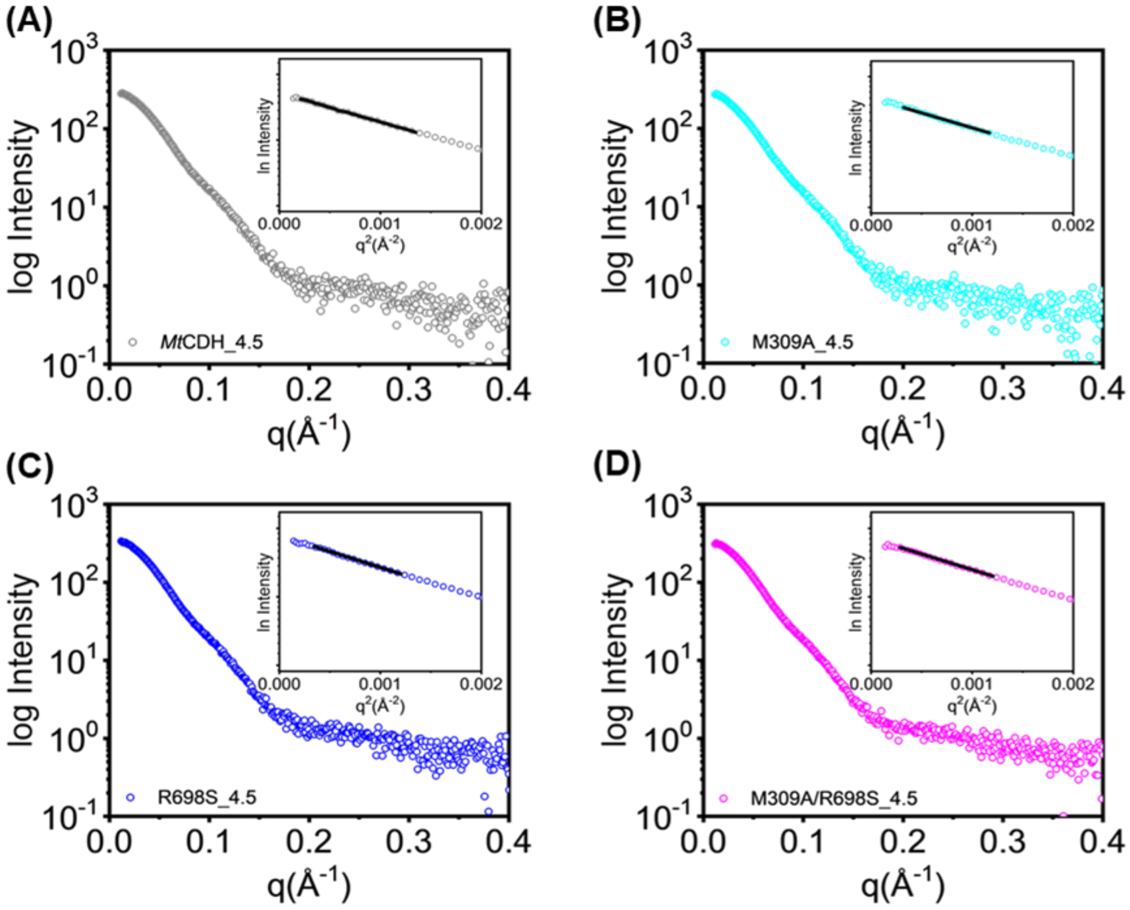

Figure S4: SAXS scattering profiles and the Guinier plots (insets) for wild-type *Mt*CDH (**A**), M309A (**B**), R698S (**C**) and M309A/R698S (**D**) at a pH of 4.5.

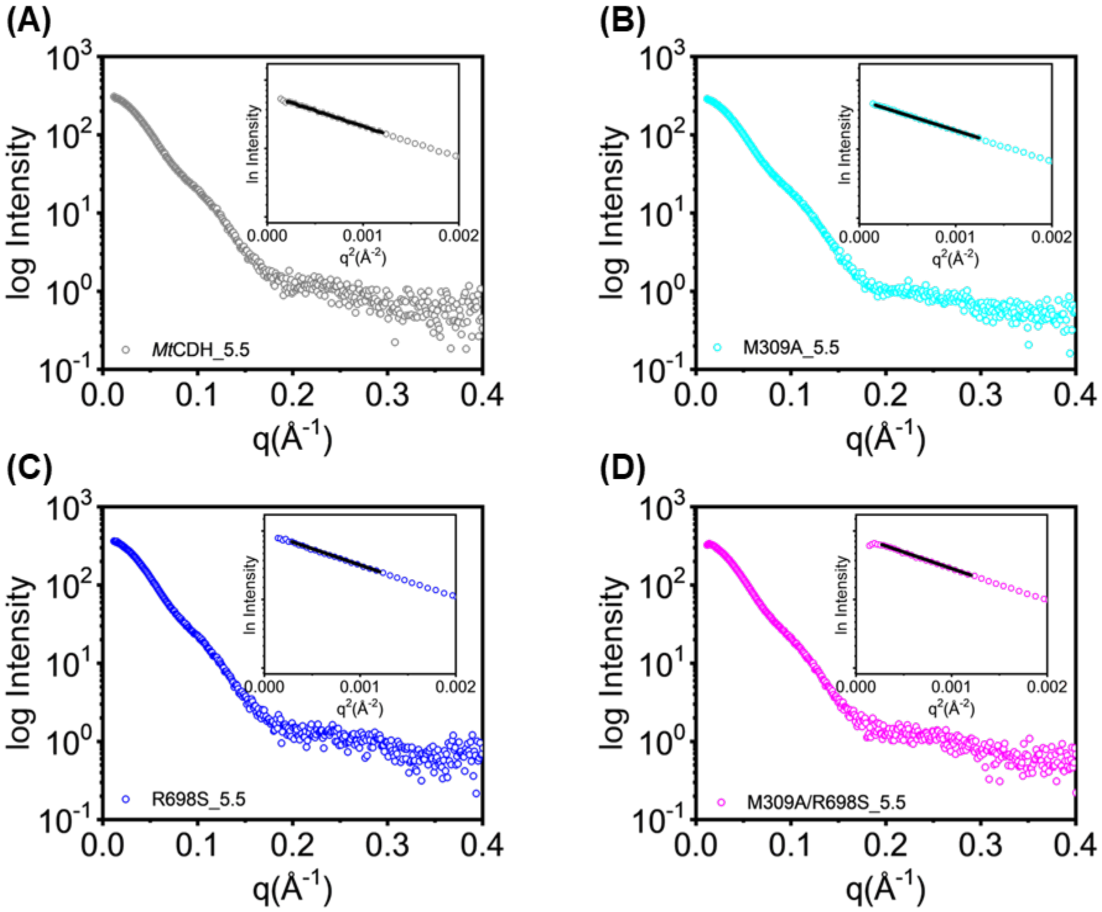

Figure S5: SAXS scattering profiles and the Guinier plots (insets) for wild-type *Mt*CDH (**A**), M309A (**B**), R698S (**C**) and M309A/R698S (**D**) at a pH of 5.5.

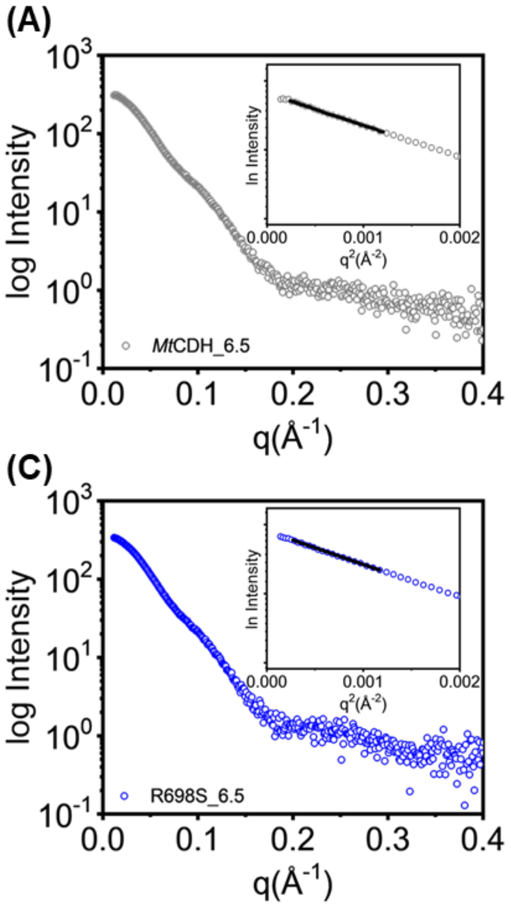

Figure S6: SAXS scattering profiles and the Guinier plots (insets) for wild-type *Mt*CDH (**A**), M309A (**B**), R698S (**C**) and M309A/R698S (**D**) at a pH of 6.5.

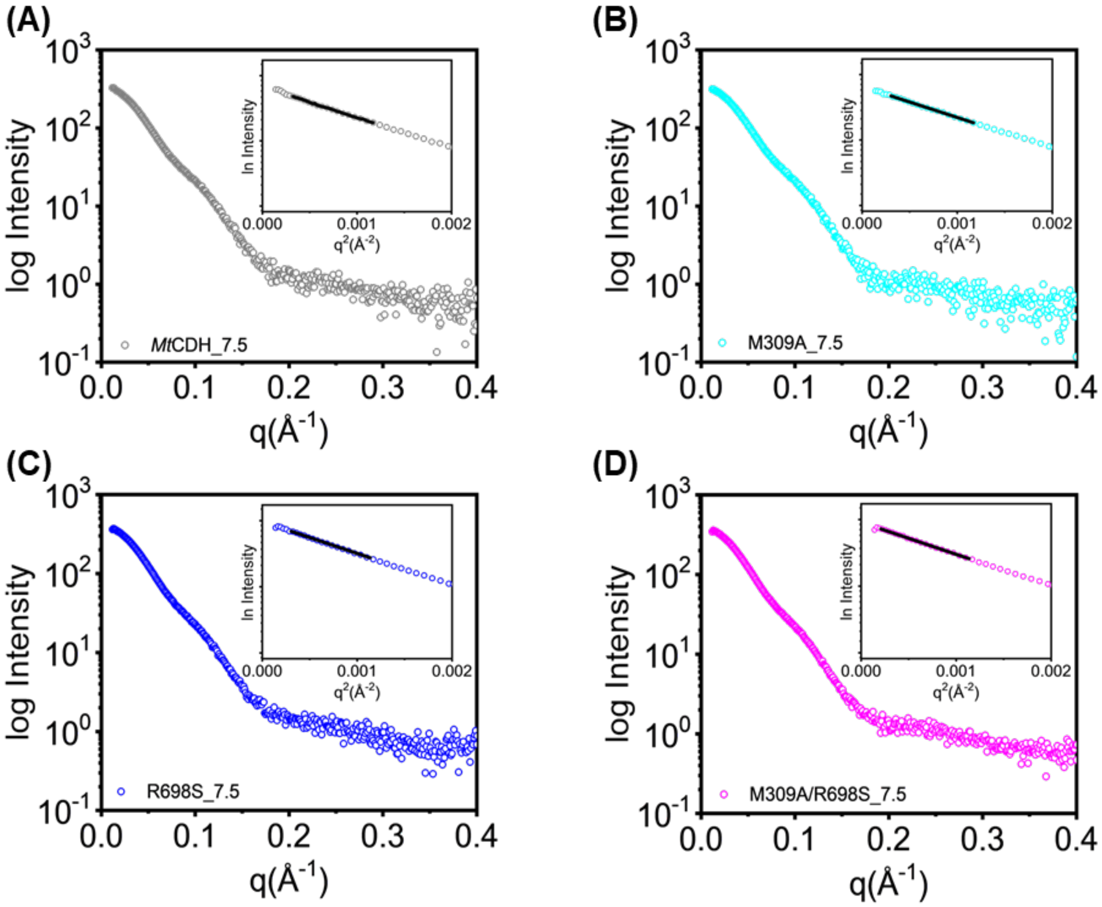

Figure S7: SAXS scattering profiles and the Guinier plots (insets) for wild-type *Mt*CDH (**A**), M309A (**B**), R698S (**C**) and M309A/R698S (**D**) at a pH of 7.5.

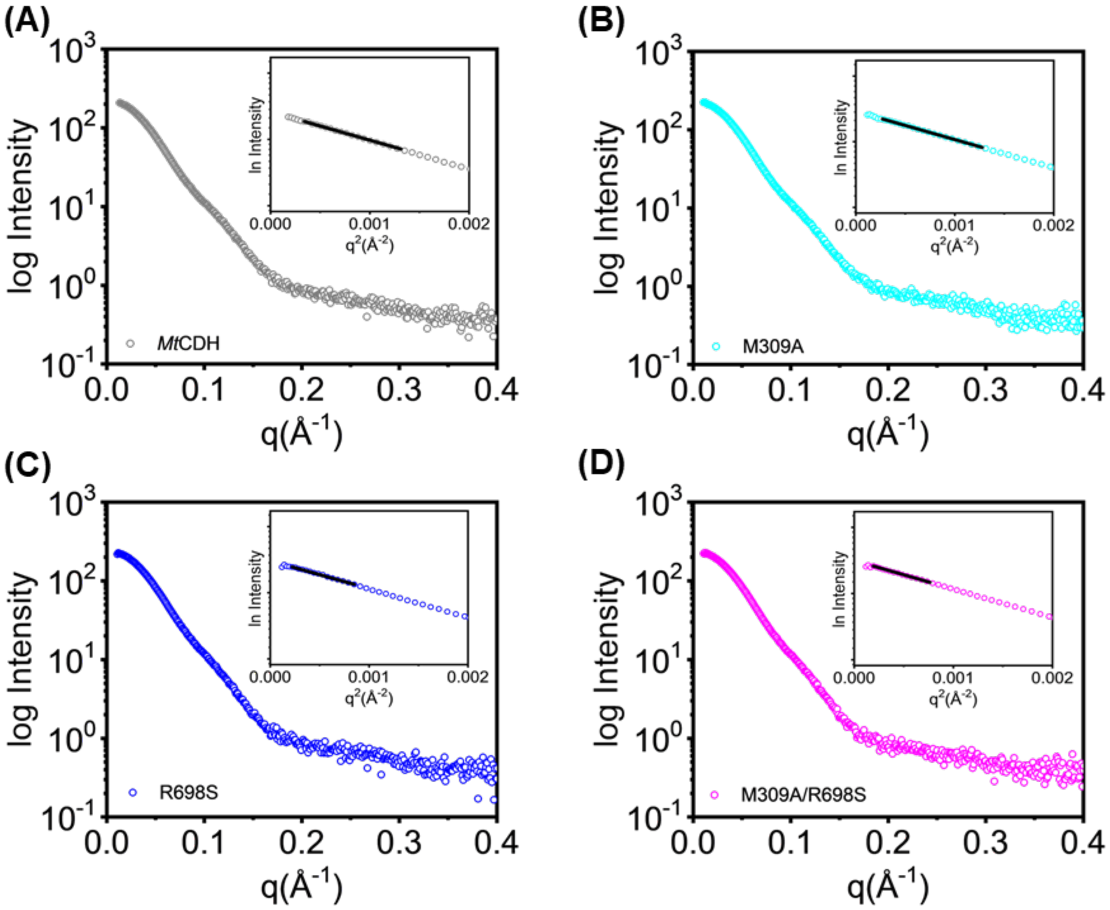

Figure S8: SAXS scattering profiles and the Guinier plots (insets) for wild-type *Mt*CDH (**A**), M309A (**B**), R698S (**C**) and M309A/R698S (**D**) at pH 5.5 without $CaCl_2$.

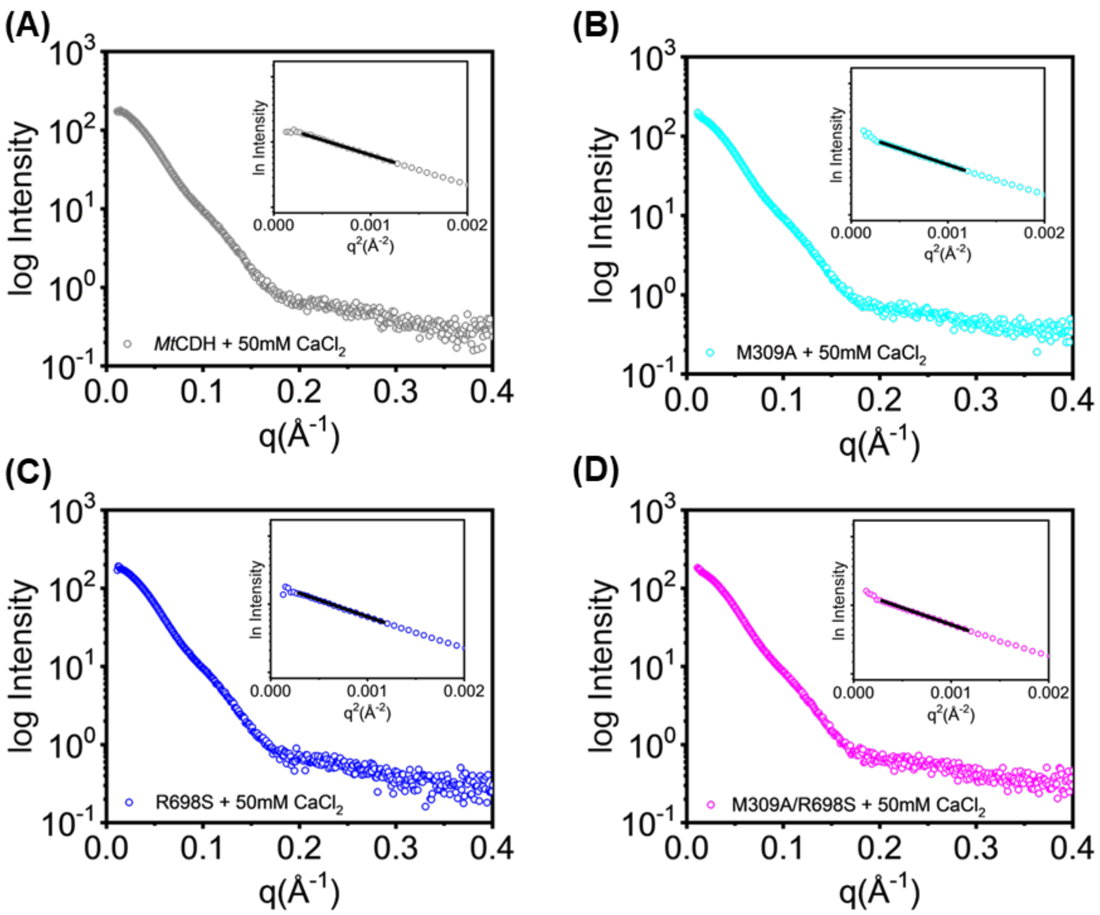

Figure S9: SAXS scattering profiles and the Guinier plots (insets) for wild-type *Mt*CDH (**A**), M309A (**B**), R698S (**C**) and M309A/R698S (**D**) at pH 5.5 in the presence of 50 mM $CaCl_2$.

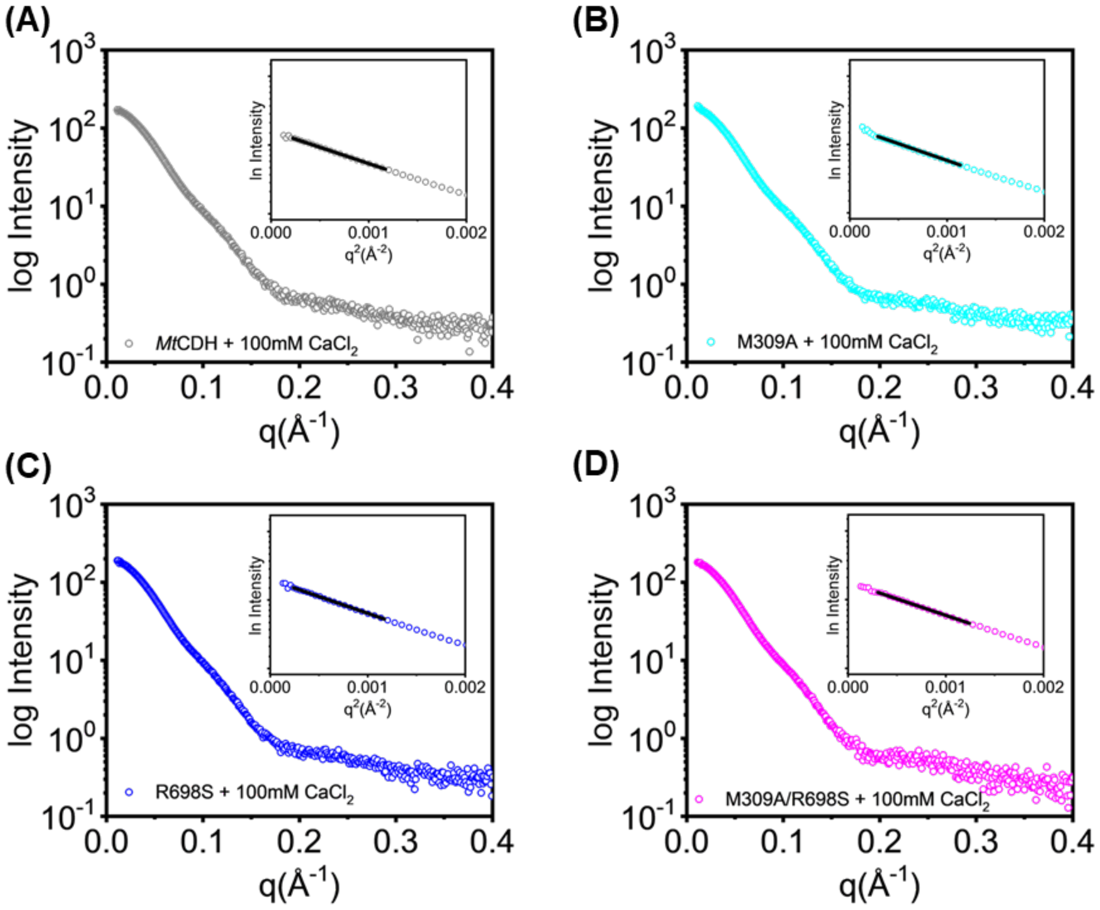

Figure S10: SAXS scattering profiles and the Guinier plots (insets) for wild-type *Mt*CDH (**A**), M309A (**B**), R698S (**C**) and M309A/R698S (**D**) at pH 5.5 in the presence of 100 mM $CaCl_2$.

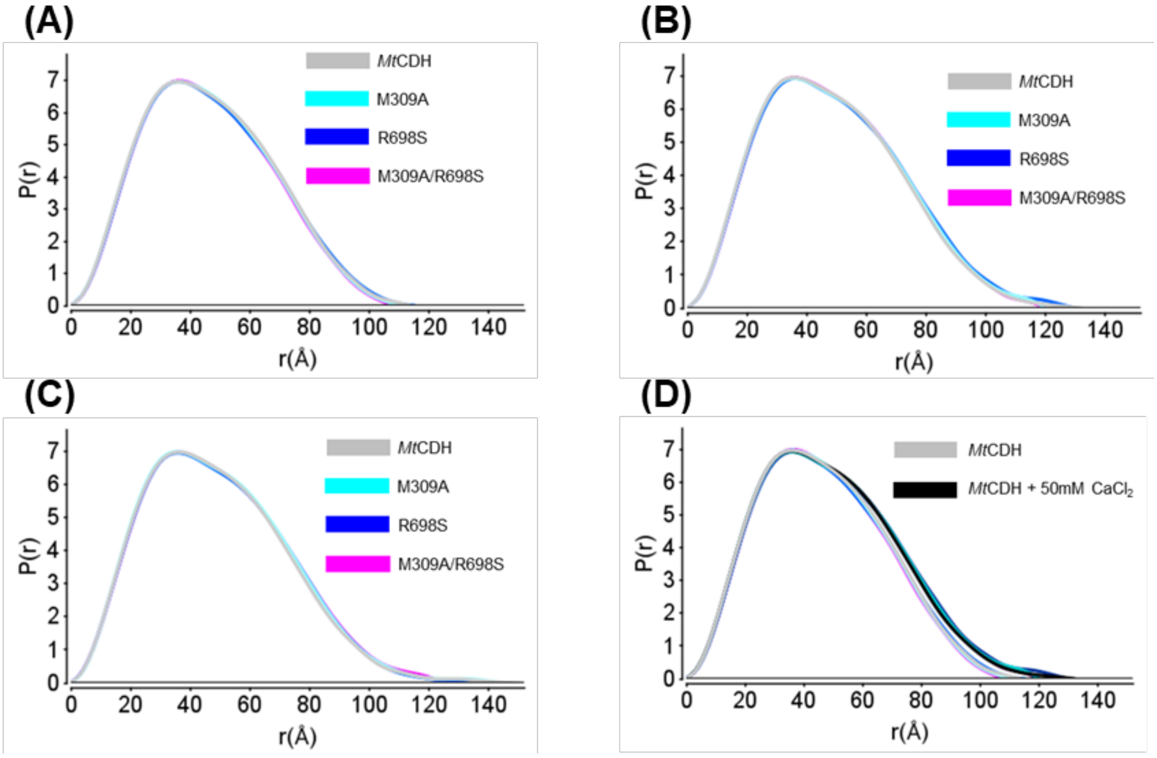

Figure S11: Effect of $CaCl_2$ addition on wild-type *Mt*CDH and all its variants at a pH of 5.5 without $CaCl_2$ **(A),** and at a concentration of 50 mM $CaCl_2$ **(B)** or 100 mM $CaCl_2$ **(C),** respectively. The measured scattering curves (**Figures S8, S9, S10**) were used to calculate the P(r) functions. **(D)** Comparisons of the pair-distance distribution functions with and without the addition of bivalent Ca^{2+} ions.

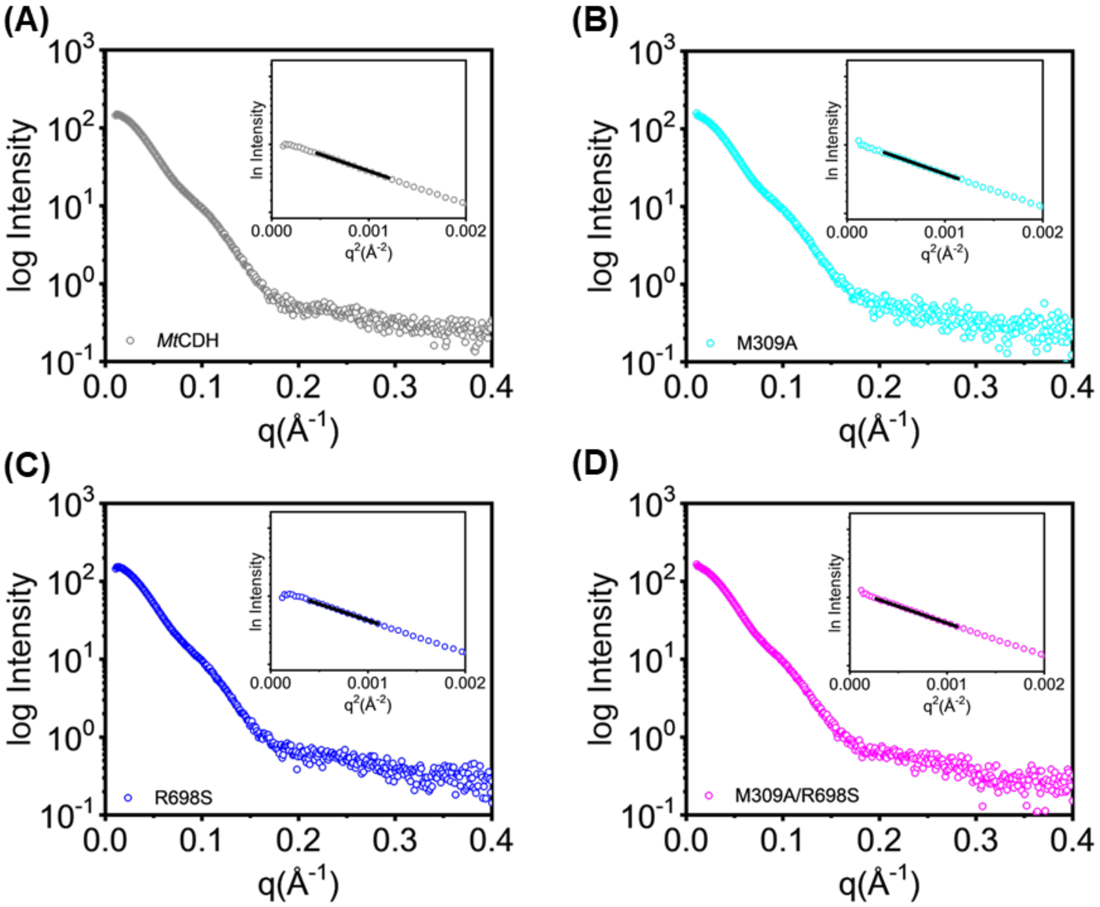

Figure S12: SAXS scattering profiles and the Guinier plots (insets) for wild-type *Mt*CDH (**A**), M309A (**B**), R698S (**C**) and M309A/R698S (**D**) at pH 6.5 without $CaCl_2$.

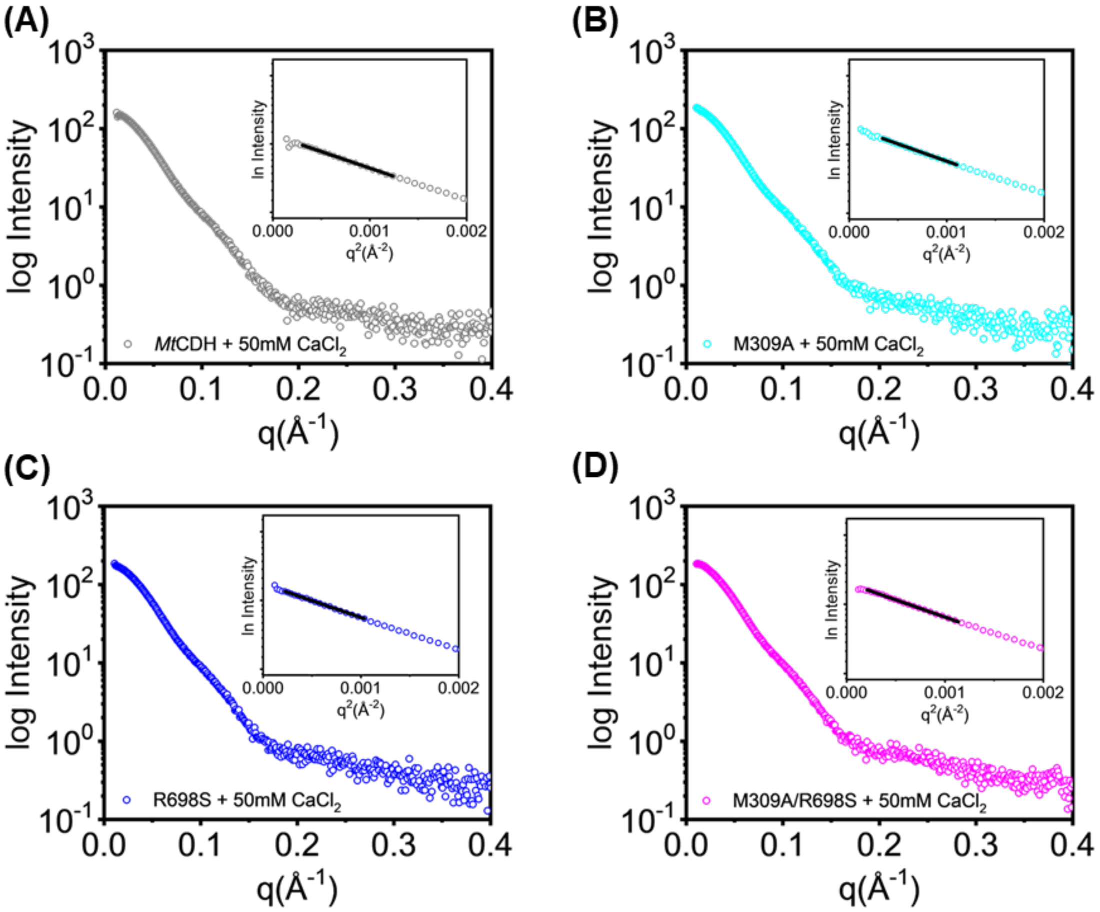

Figure S13: SAXS scattering profiles and the Guinier plots (insets) for wild-type *Mt*CDH (**A**), M309A (**B**), R698S (**C**) and M309A/R698S (**D**) at pH 6.5 with the addition of 50 mM $CaCl_2$.

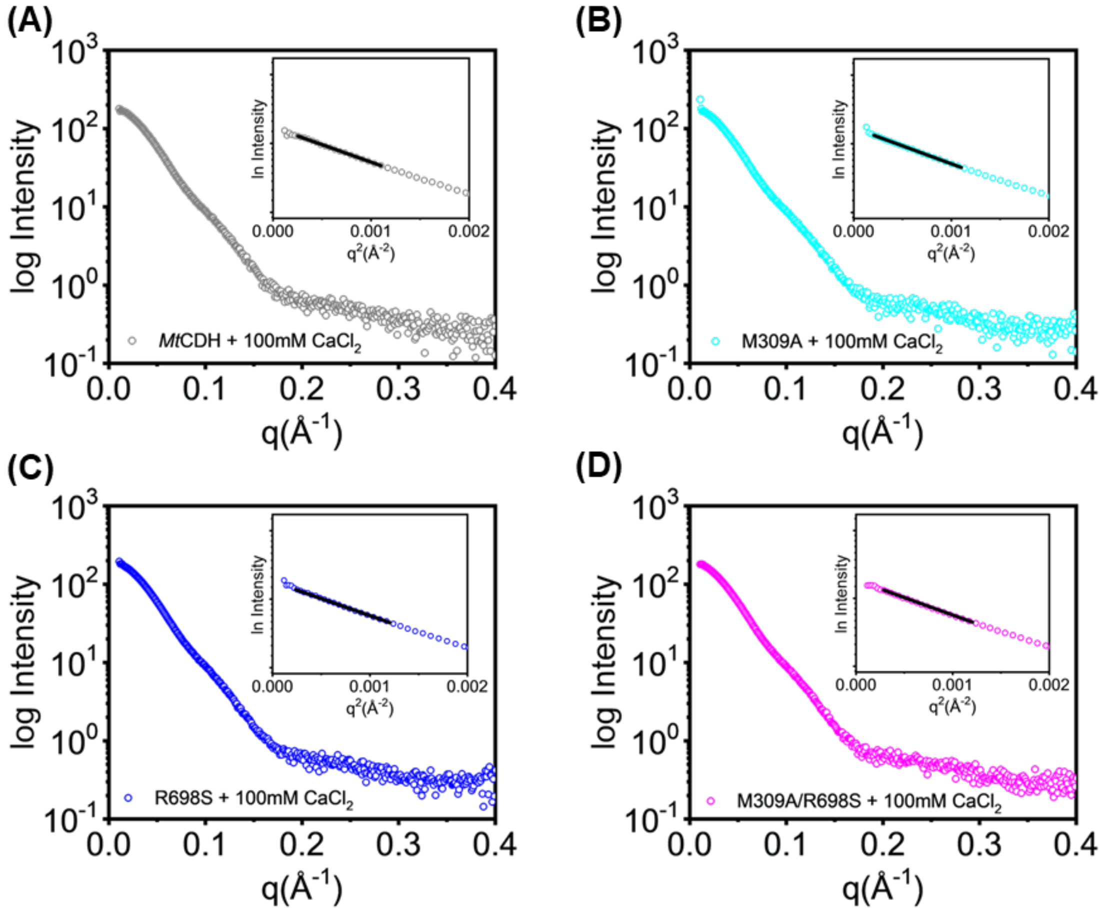

Figure S14: SAXS scattering profiles and the Guinier plots (insets) for wild-type *Mt*CDH (**A**), M309A (**B**), R698S (**C**) and M309A/R698S (**D**) at pH 6.5 with the addition of 100 mM $CaCl_2$.

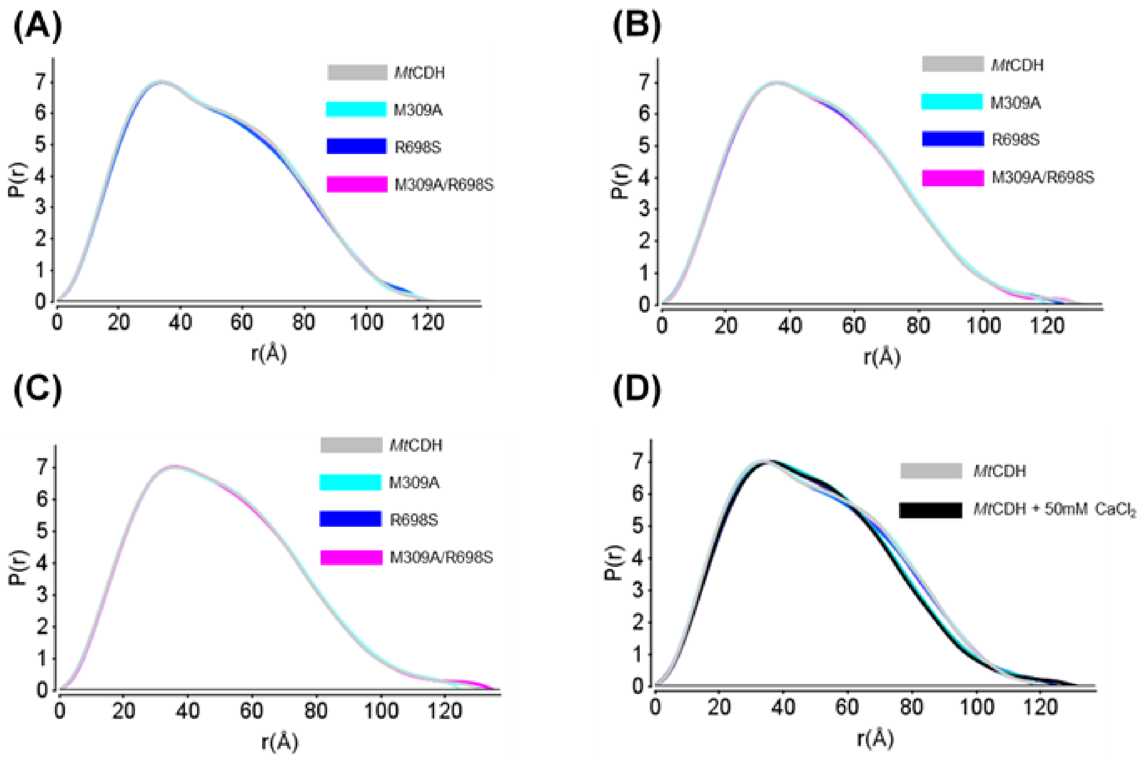

Figure S15: Pair-distance-distribution function, calculated from the measured data (**Figures S12, S13, S14),** of wild-type *Mt*CDH and all its variants for pH 6.5 without $CaCl_2$ **(A)**, and at a concentration of 50 mM $CaCl_2$ **(B)**, or 100 mM $CaCl_2$ **(C)**. **(D)** Comparisons of the pair-distance distribution functions with and without the addition of bivalent Ca^{2+} ions.

Table S1A: FAD and heme *b* reduction rates for wild-type *Mt*CDH and its variants at different pH values – sorted by pH.

Condition	Enzyme	FAD reduction rate (s^{-1})	Heme *b* reduction rate (s^{-1}) (IET)
**pH 3.5**	*Mt*CDH	10.5 ± 0.2	0.61 ± 0.001
	M309A	10.4 ± 1.5	0.06 ± 0.002
	R698S	12.4 ± 0.3	0.06 ± 0.001
	M309A/R698S	13.5 ± 0.6	0.04 ± 0.001
**pH 4.5**	*Mt*CDH	12.3 ± 0.8	0.64 ± 0.002
	M309A	9.7 ± 0.7	0.04 ± 0.001
	R698S	14.0 ± 0.6	0.03 ± 0.001
	M309A/R698S	8.6 ± 0.5	0.04 ± 0.003
**pH 5.5**	*Mt*CDH	14.4 ± 0.2	0.29 ± 0.001
	M309A	10.5 ± 0.4	0.03 ± 0.001
	R698S	14.6 ± 1.0	0.02 ± 0.002
	M309A/R698S	12.0 ± 0.6	0.03 ± 0.004
**pH 6.5**	*Mt*CDH	22.5 ± 0.5	0.07 ± 0.004
	M309A	12.2 ± 0.4	0.03 ± 0.001
	R698S	26.1 ± 1.5	0.02 ± 0.003
	M309A/R698S	15.7 ± 0.4	0.02 ± 0.004
**pH 7.5**	*Mt*CDH	30.6 ± 1.2	0.03 ± 0.001
	M309A	14.8 ± 0.6	0.01 ± 0.001
	R698S	34.8 ± 1.7	0.04 ± 0.007
	M309A/R698S	18.3 ± 0.8	0.03 ± 0.002

Table S1B: FAD and heme *b* reduction rates for wild-type *Mt*CDH and is variants at different pH values – sorted by enzyme variant.

Enzyme	Condition	FAD reduction rate (s^{-1})	Heme *b* reduction rate (s^{-1}) (IET)
***Mt*CDH**	pH 3.5	10.5 ± 0.2	0.61 ± 0.001
	pH 4.5	12.3 ± 0.8	0.64 ± 0.002
	pH 5.5	14.4 ± 0.2	0.29 ± 0.001
	pH 6.5	22.5 ± 0.5	0.07 ± 0.004
	pH 7.5	30.6 ± 1.2	0.03 ± 0.001
**M309A**	pH 3.5	10.4 ± 1.5	0.06 ± 0.002
	pH 4.5	9.7 ± 0.7	0.04 ± 0.001
	pH 5.5	10.5 ± 0.4	0.03 ± 0.001
	pH 6.5	12.2 ± 0.4	0.03 ± 0.001
	pH 7.5	14.8 ± 0.6	0.01 ± 0.001
**R698S**	pH 3.5	12.4 ± 0.3	0.06 ± 0.001
	pH 4.5	14.0 ± 0.6	0.03 ± 0.001
	pH 5.5	14.6 ± 1.0	0.02 ± 0.002
	pH 6.5	26.1 ± 1.5	0.02 ± 0.003
	pH 7.5	34.8 ± 1.7	0.04 ± 0.007
**M309A/R698S**	pH 3.5	13.5 ± 0.6	0.04 ± 0.001
	pH 4.5	8.6 ± 0.5	0.04 ± 0.003
	pH 5.5	12.0 ± 0.6	0.03 ± 0.004
	pH 6.5	15.7 ± 0.4	0.02 ± 0.004
	pH 7.5	18.3 ± 0.8	0.03 ± 0.002

Table S2A: FAD and heme *b* reduction rates for wild-type *Mt*CDH and its variants at two pH values and two $CaCl_2$ concentrations – sorted by condition.

Condition	Enzyme	FAD reduction (s^{-1})	Heme *b* reduction (s^{-1}) (IET)
**pH 5.5**	*Mt*CDH	14.5 ± 0.1	0.14 ± 0.003
	M309A	9.8 ± 0.9	0.03 ± 0.001
	R698S	17.1 ± 0.6	0.03 ± 0.003
	M309A/R698S	10.3 ± 0.9	0.02 ± 0.001
**+ 50 mM $CaCl_2$**	*Mt*CDH	17.7 ± 0.6	2.01 ± 0.008
	M309A	8.5 ± 0.8	0.21 ± 0.002
	R698S	18.8 ± 0.3	0.07 ± 0.001
	M309A/R698S	8.7 ± 0.7	0.02 ± 0.001
**+ 100 mM $CaCl_2$**	*Mt*CDH	22.5 ± 0.3	2.33 ± 0.009
	M309A	9.3 ± 0.3	0.26 ± 0.001
	R698S	22.2 ± 0.4	0.09 ± 0.001
	M309A/R698S	7.3 ± 0.9	0.03 ± 0.001
**pH 6.5**	*Mt*CDH	19.1 ± 0.7	0.06 ± 0.002
	M309A	8.7 ± 0.5	0.03 ± 0.001
	R698S	23.8 ± 1.5	0.02 ± 0.008
	M309A/R698S	7.5 ± 0.5	0.06 ± 0.007
**+ 50 mM $CaCl_2$**	*Mt*CDH	38.1 ± 1.8	1.86 ± 0.002
	M309A	11.3 ± 0.6	0.18 ± 0.001
	R698S	33.9 ± 1.8	0.08 ± 0.001
	M309A/R698S	12.4 ± 0.8	0.03 ± 0.001
**+ 100 mM $CaCl_2$**	*Mt*CDH	38.6 ± 1.6	2.10 ± 0.007
	M309A	10.9 ± 0.9	0.17 ± 0.001
	R698S	45.9 ± 1.7	0.09 ± 0.001
	M309A/R698S	11.5 ± 0.3	0.04 ± 0.001

Table S2B: FAD and heme *b* reduction rates for wild-type *Mt*CDH and its variants at two pH values and two $CaCl_2$ concentrations – sorted by enzyme variant.

Enzyme	Condition	FAD reduction rate (s^{-1})	Heme *b* reduction rate (s^{-1}) (IET)
***Mt*CDH**	pH 5.5	14.5 ± 0.1	0.14 ± 0.003
	+ 50 mM	17.7 ± 0.6	2.01 ± 0.008
	+ 100 mM	22.5 ± 0.3	2.33 ± 0.009
	pH 6.5	19.1 ± 0.7	0.06 ± 0.002
	+ 50 mM	38.1 ± 1.8	1.86 ± 0.002
	+ 100 mM	38.6 ± 1.6	2.10 ± 0.007
**M309A**	pH 5.5	9.8 ± 0.9	0.03 ± 0.001
	+ 50 mM	8.5 ± 0.8	0.21 ± 0.002
	+ 100 mM	9.3 ± 0.3	0.26 ± 0.001
	pH 6.5	8.7 ± 0.5	0.03 ± 0.001
	+ 50 mM	11.3 ± 0.6	0.18 ± 0.001
	+ 100 mM	10.9 ± 0.9	0.17 ± 0.001
**R698S**	pH 5.5	17.1 ± 0.6	0.03 ± 0.003
	+ 50 mM	18.8 ± 0.3	0.07 ± 0.001
	+ 100 mM	22.2 ± 0.4	0.09 ± 0.001
	pH 6.5	23.8 ± 1.5	0.02 ± 0.008
	+ 50 mM	33.9 ± 1.8	0.08 ± 0.001
	+ 100 mM	45.9 ± 1.7	0.09 ± 0.001
**M309A/R698S**	pH 5.5	10.3 ± 0.9	0.02 ± 0.001
	+ 50 mM	8.7 ± 0.7	0.02 ± 0.001
	+ 100 mM	7.3 ± 0.9	0.03 ± 0.001
	pH 6.5	7.5 ± 0.5	0.06 ± 0.007
	+ 50 mM	12.4 ± 0.8	0.03 ± 0.001
	+ 100 mM	11.5 ± 0.3	0.04 ± 0.001

Table S3A: SAXS results of wild-type *Mt*CDH and its variants. The molecular weight (MW) identified by MALS and SAXS (q region specified in Table S5), the radius of gyration (Rg) and the maximal diameter (D_{max}) as determined by SAXS are shown for different pH-values – sorted by pH.

Condition	Enzyme	MW (kDa) MALS	MW (kDa) SAXS	Rg (Å)	D_{max} (Å)
**pH 3.5**	*Mt*CDH	97	104	37.3 ± 0.2	145
	M309A	96	103	36.2 ± 0.2	127
	R698S	97	106	39.6 ± 0.4	145
	M309A/R698S	98	106	40.2 ± 0.7	147
**pH 4.5**	*Mt*CDH	92	93	36.6 ± 0.3	120
	M309A	93	95	36.1 ± 0.7	125
	R698S	94	97	37.4 ± 0.4	126
	M309A/R698S	94	96	36.8 ± 0.4	125
**pH 5.5**	*Mt*CDH	92	92	37.2 ± 0.4	125
	M309A	93	92	37.0 ± 0.3	120
	R698S	93	96	37.5 ± 0.4	121
	M309A/R698S	93	95	37.6 ± 0.3	120
**pH 6.5**	*Mt*CDH	93	92	37.5 ± 0.3	118
	M309A	94	91	37.4 ± 0.5	119
	R698S	94	95	37.8 ± 0.3	117
	M309A/R698S	95	96	37.6 ± 0.4	124
**pH 7.5**	*Mt*CDH	93	93	37.8 ± 0.4	120
	M309A	93	94	37.9 ± 0.4	121
	R698S	95	96	38.3 ± 0.3	126
	M309A/R698S	95	97	38.1 ± 0.3	127

Table S3B: SAXS results of wild-type *Mt*CDH and its variants. The molecular weight (MW) identified by MALS and SAXS (q region specified in Table S5), the radius of gyration (Rg) and the maximal diameter (D_{max}) as determined by SAXS are shown for different pH-values – sorted by enzyme variant.

Enzyme	Condition	MW (kDa)	MW (kDa)	Rg (Å)	D_{max} (Å)
		MALS	SAXS		
***Mt*CDH**	pH 3.5	97	104	37.3 ± 0.2	145
	pH 4.5	92	93	36.6 ± 0.3	120
	pH 5.5	92	92	37.2 ± 0.4	125
	pH 6.5	93	92	37.5 ± 0.3	118
	pH 7.5	93	93	37.8 ± 0.4	120
**M309A**	pH 3.5	96	103	36.2 ± 0.2	127
	pH 4.5	93	95	36.1 ± 0.7	125
	pH 5.5	93	92	37.0 ± 0.3	120
	pH 6.5	94	91	37.4 ± 0.5	119
	pH 7.5	93	94	37.9 ± 0.4	121
**R698S**	pH 3.5	97	106	39.6 ± 0.4	145
	pH 4.5	94	97	37.4 ± 0.4	126
	pH 5.5	93	96	37.5 ± 0.4	121
	pH 6.5	95	95	37.8 ± 0.3	117
	pH 7.5	94	96	38.3 ± 0.3	126
**M309A/R698S**	pH 3.5	98	106	40.2 ± 0.7	147
	pH 4.5	94	96	36.8 ± 0.4	125
	pH 5.5	93	95	37.6 ± 0.3	120
	pH 6.5	95	96	37.6 ± 0.4	124
	pH 7.5	95	97	38.1 ± 0.3	127

Table S4A: Prediction of the P(r) function from CDH models for the pH map. The best-fitting multi-state models of the measured SAXS profiles are given for wild-type *Mt*CDH and all its variants. The Rg values and the weight of each conformer (w) are shown – sorted by pH.

**Condition**	**Enzyme**	**Single-state**	**Two-state**	**Model 1**		**Model 2**	
		χ^2	χ^2	**R_g (Å)**	**w (%)**	**R_g (Å)**	**w (%)**
**pH 3.5**	*Mt*CDH	1.48	1.17	32.6	67	41.3	33
	M309A	1.49	1.03	32.4	62	40.8	38
	R698S	1.75	1.45	33.5	53	40.9	47
	M309A/R698S	1.59	1.44	33.1	48	40.8	52
**pH 4.5**	*Mt*CDH	1.47	1.22	34.9	87	47.9	13
	M309A	1.23	1.09	35.1	85	51.0	15
	R698S	1.43	1.24	34.7	72	41.9	28
	M309A/R698S	1.41	1.24	34.5	72	42.0	28
**pH 5.5**	*Mt*CDH	1.25	1.21	36.5	89	57.2	11
	M309A	1.35	1.21	36.1	91	53.3	9
	R698S	1.11	1.07	35.9	87	44.7	13
	M309A/R698S	1.44	1.39	36.0	88	46.7	12
**pH 6.5**	*Mt*CDH	1.49	1.44	36.7	95	48.7	5
	M309A	1.25	1.17	36.8	89	58.0	11
	R698S	1.23	1.17	36.7	82	40.5	18
	M309A/R698S	1.20	1.15	36.1	87	45.0	13
**pH 7.5**	*Mt*CDH	1.37	1.33	37.0	90	58.8	10
	M309A	1.40	1.35	36.7	93	48.9	7
	R698S	1.32	1.27	36.0	81	44.6	19
	M309A/R698S	1.26	1.14	36.7	92	48.4	8

Table S4B: Prediction of the P(r) function from CDH models for the pH map. The best-fitting multi-state models of the measured SAXS profiles are given for wild-type *Mt*CDH and all its variants. The Rg values and the weight of each conformer (w) are shown – sorted by enzyme variant.

Enzyme	Condition	Single-state	Two-state	Model 1		Model 2	
		χ^2	χ^2	R_g (Å)	w (%)	R_g (Å)	w (%)
	pH 3.5	1.48	1.17	32.6	67	41.3	33
	pH 4.5	1.47	1.22	34.9	87	47.9	13
***Mt*CDH**	pH 5.5	1.25	1.21	36.5	89	57.2	11
	pH 6.5	1.49	1.44	36.7	95	48.7	5
	pH 7.5	1.37	1.33	37.0	90	58.8	10
	pH 3.5	1.49	1.03	32.4	62	40.8	38
	pH 4.5	1.23	1.09	35.1	85	51.0	15
**M309A**	pH 5.5	1.35	1.21	36.1	91	53.3	9
	pH 6.5	1.25	1.17	36.8	89	58.0	11
	pH 7.5	1.40	1.35	36.7	93	48.9	7
	pH 3.5	1.75	1.45	33.5	53	40.9	47
	pH 4.5	1.43	1.24	34.7	72	41.9	28
**R698S**	pH 5.5	1.11	1.07	35.9	87	44.7	13
	pH 6.5	1.23	1.17	36.7	82	40.5	18
	pH 7.5	1.32	1.27	36.0	81	44.6	19
	pH 3.5	1.59	1.44	33.1	48	40.8	52
	pH 4.5	1.41	1.24	34.5	72	42.0	28
**M309A/R698S**	pH 5.5	1.44	1.39	36.0	88	46.7	12
	pH 6.5	1.20	1.15	36.1	87	45.0	13
	pH 7.5	1.26	1.14	36.7	92	48.4	8

Table S5: q ranges in the Guinier plots, which were used to analyze the molecular weight from the SAXS data for the pH profile.

Enzyme	Condition	MW (kDa)	Q range
***Mt*CDH**	pH 3.5	104	0.01900 – 0.03573
	pH 4.5	93	0.01495 – 0.03674
	pH 5.5	92	0.01546 – 0.03471
	pH 6.5	92	0.01460 – 0.03522
	pH 7.5	93	0.01851 – 0.03421
**M309A**	pH 3.5	103	0.02154 – 0.03674
	pH 4.5	95	0.01799 – 0.03421
	pH 5.5	92	0.01292 – 0.03522
	pH 6.5	91	0.01951 – 0.03471
	pH 7.5	94	0.01748 – 0.03421
**R698S**	pH 3.5	106	0.01596 – 0.03370
	pH 4.5	97	0.01850 – 0.03471
	pH 5.5	96	0.01698 – 0.03471
	pH 6.5	95	0.01647 – 0.03421
	pH 7.5	96	0.01748 – 0.03337
**M309A/R698S**	pH 3.5	106	0.01647 – 0.03269
	pH 4.5	96	0.01698 – 0.03522
	pH 5.5	95	0.01647 – 0.03471
	pH 6.5	96	0.01799 – 0.03421
	pH 7.5	97	0.01444 – 0.03370

Table S6A: SAXS measurements elucidating the effect of Ca^{2+} ions. Obtained molecular weight (MW), the radius of gyration (Rg) and the maximal diameters (D_{max}) for wild-type *Mt*CDH and its variants at pH 5.5 and different $CaCl_2$ concentrations as determined by MALS and SAXS (q region specified in Table S10) are shown – sorted by pH.

**Condition**	**Enzyme**	**MW (kDa)**	**MW (kDa)**	**Rg (Å)**	**D_{max} (Å)**
		**MALS**	**SAXS**		
**pH 5.5**	*Mt*CDH	100	98	36.3 ± 0.3	114
	M309A	100	99	35.8 ± 0.3	111
	R698S	101	101	36.0 ± 0.3	115
	M309A/R698S	101	102	35.7 ± 0.2	108
**+ 50 mM**	*Mt*CDH	102	101	37.7 ± 0.4	132
	M309A	102	101	38.8 ± 0.5	120
	R698S	103	104	38.1 ± 0.4	130
	M309A/R698S	103	104	37.9 ± 0.4	120
**+ 100 mM**	*Mt*CDH	104	103	38.0 ± 0.3	150
	M309A	103	102	38.6 ± 0.4	145
	R698S	104	105	38.7 ± 0.4	135
	M309A/R698S	103	106	38.6 ± 0.4	125

Table S6B: SAXS measurements elucidating the effect of Ca^{2+} ions. Obtained molecular weight (MW), the radius of gyration (Rg) and the maximal diameters (D_{max}) for wild-type *Mt*CDH and its variants at pH 5.5 and different $CaCl_2$ concentrations as determined by MALS and SAXS (q region specified in Table S10) are shown – sorted by enzyme variant.

**Enzyme**	**Condition**	**MW (kDa)**	**MW (kDa)**	**Rg (Å)**	**D_{max} (Å)**
		**MALS**	**SAXS**		
	pH 5.5	100	98	36.3 ± 0.3	114
***Mt*CDH**	+ 50 mM	102	101	37.7 ± 0.4	132
	+100 mM	104	103	38.0 ± 0.3	150
	pH 5.5	100	99	35.8 ± 0.3	111
**M309A**	+ 50 mM	102	101	38.8 ± 0.5	120
	+100 mM	103	102	38.6 ± 0.4	145
	pH 5.5	101	101	36.0 ± 0.3	115
**R698S**	+ 50 mM	103	104	38.1 ± 0.4	130
	+100 mM	104	105	38.7 ± 0.4	135
	pH 5.5	101	102	35.7 ± 0.2	108
**M398A/R698S**	+ 50 mM	103	104	37.9 ± 0.4	120
	+100 mM	103	106	38.6 ± 0.4	125

Table S7A: Modelling parameters Rg and the weight of each conformer (w) of wild-type *Mt*CDH and all its variants at different $CaCl_2$ concentrations. Results from the best-fitting multi-state models are shown – sorted by condition.

Condition	Enzyme	Single-state	Two-state	Model 1		Model 2	
		χ^2	χ^2	R_g (Å)	w (%)	R_g (Å)	w (%)
**pH 5.5**	*Mt*CDH	2.32	1.25	36.0	52	34.7	48
	M309A	2.29	1.16	37.4	44	33.2	56
	R698S	1.93	1.25	37.3	55	33.0	45
	M309A/R698S	2.83	1.43	37.3	46	33.5	54
**+ 50 mM**	*Mt*CDH	2.24	1.27	38.4	58	33.3	42
	M309A	1.80	1.01	39.7	43	34.7	57
	R698S	2.14	1.40	40.2	41	33.4	59
	M309A/R698S	2.24	1.48	38.4	63	33.4	37
**+ 100 mM**	*Mt*CDH	1.83	1.03	38.8	56	33.2	44
	M309A	2.04	1.10	38.5	57	33.3	43
	R698S	1.89	1.18	38.8	64	32.1	36
	M309A/R698S	2.05	1.50	37.7	56	33.0	44

Table S7B: Modelling parameters Rg and w of wild-type *Mt*CDH and all its variants at different $CaCl_2$ concentrations. Results from the best-fitting multi-state models are shown – sorted by enzyme variant.

**Enzyme**	**Condition**	**Single-state**	**Two-state**	**Model 1**		**Model 2**	
		χ^2	χ^2	**R_g (Å)**	**w (%)**	**R_g (Å)**	**w (%)**
	pH 5.5	2.32	1.25	36.0	52	34.7	48
***Mt*CDH**	+ 50 mM	2.24	1.27	38.4	58	33.3	42
	+ 100 mM	1.83	1.03	38.8	56	33.2	44
	pH 5.5	2.29	1.16	37.4	44	33.2	56
**M309A**	+ 50 mM	1.80	1.01	39.7	43	34.7	57
	+ 100 mM	2.04	1.10	38.5	57	33.3	43
	pH 5.5	1.93	1.25	37.3	55	33.0	45
**R698S**	+ 50 mM	2.14	1.40	40.2	41	33.4	59
	+ 100 mM	1.89	1.18	38.8	64	32.1	36
	pH 5.5	2.83	1.43	37.3	46	33.5	54
**M309A/R698S**	+ 50 mM	2.24	1.48	38.4	63	33.4	37
	+ 100 mM	2.05	1.50	37.7	56	33.0	44

Table S8A: SAXS measurements elucidating the effect of Ca^{2+} ions at pH 6.5. The calculated molecular weight (MW), the radius of gyration (Rg) and the maximal diameters (D_{max}) determined by MALS and SAXS (q region specified in Table S10) are shown – sorted by pH.

**Condition**	**Enzyme**	**MW (kDa)**	**MW (kDa)**	**Rg (Å)**	**D_{max} (Å)**
		**MALS**	**SAXS**		
**pH 6.5**	*Mt*CDH	100	102	38.3 ± 0.4	122
	M309A	100	100	38.5 ± 0.5	120
	R698S	99	101	38.5 ± 0.4	118
	M309A/R698S	100	101	38.5 ± 0.3	120
**+ 50 mM**	*Mt*CDH	102	105	38.5 ± 0.4	131
	M309A	103	103	38.7 ± 0.5	120
	R698S	102	106	38.5 ± 0.4	125
	M309A/R698S	102	105	38.6 ± 0.3	130
**+ 100 mM**	*Mt*CDH	103	106	38.9 ± 0.4	132
	M309A	104	106	39.3 ± 0.4	125
	R698S	104	108	39.3 ± 0.3	120
	M309A/R698S	104	107	39.5 ± 0.3	135

Table S8B: SAXS measurements elucidating the effect of Ca^{2+} ions at pH 6.5. The calculated molecular weight (MW), the radius of gyration (Rg) and the maximal diameters (D_{max}) determined by MALS and SAXS (q region specified in Table S10) are shown – sorted by enzyme variant.

Enzyme	Condition	MW (kDa)	MW (kDa)	Rg (Å)	D_{max} (Å)
		MALS	SAXS		
***Mt*CDH**	pH 6.5	100	102	38.3 ± 0.4	122
	+ 50 mM	102	105	38.5 ± 0.4	131
	+100 mM	103	106	38.9 ± 0.4	132
**M309A**	pH 6.5	100	100	38.5 ± 0.5	120
	+ 50 mM	103	103	38.7 ± 0.5	120
	+100 mM	104	106	39.3 ± 0.4	125
**R698S**	pH 6.5	99	101	38.5 ± 0.4	118
	+ 50 mM	102	106	38.5 ± 0.4	125
	+100 mM	104	108	39.3 ± 0.3	120
**M398A/R698S**	pH 6.5	100	101	38.5 ± 0.3	120
	+ 50 mM	102	105	38.6 ± 0.3	130
	+100 mM	104	107	39.5 ± 0.3	135

Table S9A: Best-fitting multi-state model parameters (Rg and w) at pH 6.5 at different $CaCl_2$ concentrations are shown – sorted by condition.

**Condition**	**Enzyme**	**Single-state**	**Two-state**	**Model 1**		**Model 2**	
		χ^2	χ^2	**R_g (Å)**	**w (%)**	**R_g (Å)**	**w (%)**
**pH 6.5**	*Mt*CDH	1.16	1.11	37.5	57	42.5	43
	M309A	1.21	1.15	36.8	67	38.8	33
	R698S	1.47	1.28	36.3	69	41.7	31
	M309A/R698S	1.46	1.27	34.7	55	40.4	45
**+ 50 mM**	*Mt*CDH	1.30	0.99	34.7	63	38.9	37
	M309A	1.32	1.01	32.9	37	38.9	63
	R698S	1.29	1.00	33.3	44	38.9	56
	M309A/R698S	1.31	0.96	33.1	32	38.4	68
**+ 100 mM**	*Mt*CDH	1.35	1.09	33.3	54	39.0	46
	M309A	1.59	1.26	33.3	41	38.5	59
	R698S	1.92	1.35	33.4	53	39.7	47
	M309A/R698S	1.89	1.25	31.7	36	39.1	64

Table S9B: Best-fitting multi-state model parameters (Rg and w) at pH 6.5 at different $CaCl_2$ concentrations are shown – sorted by enzyme variant.

Enzyme	Condition	Single-state	Two-state	Model 1		Model 2	
		χ^2	χ^2	R_g (Å)	w (%)	R_g (Å)	w (%)
***Mt*CDH**	pH 6.5	1.16	1.11	37.5	57	42.5	43
	+ 50 mM	1.30	0.99	34.7	63	38.9	37
	+ 100 mM	1.35	1.09	33.3	54	39.0	46
**M309A**	pH 6.5	1.21	1.15	36.8	67	38.8	33
	+ 50 mM	1.32	1.01	32.9	37	38.9	63
	+ 100 mM	1.59	1.26	33.3	41	38.5	59
**R698S**	pH 6.5	1.47	1.28	36.3	69	41.7	31
	+ 50 mM	1.29	1.00	33.3	44	38.9	56
	+ 100 mM	1.92	1.35	33.4	53	39.7	47
**M309A/R698S**	pH 6.5	1.46	1.27	34.7	55	40.4	45
	+ 50 mM	1.31	0.96	33.1	32	38.4	68
	+ 100 mM	1.89	1.25	31.7	36	39.1	64

Table S10: q ranges in the Guinier plots, which were used to analyze the molecular weight from the SAXS measurements at two pH values and two $CaCl_2$ concentrations.

Enzyme	Condition	MW (kDa)	q-range
	pH 5.5	98	0.01850 – 0.03623
	+ 50 mM	101	0.01748 – 0.03522
	+ 100 mM	103	0.01495 – 0.03421
***Mt*CDH**	pH 6.5	102	0.02306 – 0.03471
	+ 50 mM	105	0.01748 – 0.03370
	+ 100 mM	106	0.01546 – 0.03319
	pH 5.5	99	0.01647 – 0.03573
	+ 50 mM	101	0.01748 – 0.03421
	+ 100 mM	102	0.01698 – 0.03370
**M309A**	pH 6.5	100	0.01951 – 0.03370
	+ 50 mM	103	0.01850 – 0.03319
	+ 100 mM	106	0.01444 – 0.03269
	pH 5.5	101	0.01495 – 0.02914
	+ 50 mM	104	0.01698 – 0.03421
	+ 100 mM	105	0.01546 – 0.03471
**R698S**	pH 6.5	101	0.02002 – 0.03319
	+ 50 mM	106	0.01495 – 0.03218
	+ 100 mM	108	0.01546 – 0.03471
	pH 5.5	102	0.01394 – 0.02762
	+ 50 mM	104	0.01698 – 0.03421
	+ 100 mM	106	0.01748 – 0.03522
**M309A/R698S**	pH 6.5	101	0.01647 – 0.03319
	+ 50 mM	105	0.01444 – 0.03370
	+ 100 mM	107	0.01698 – 0.03471
